# SGLT2 inhibitors and atrial fibrillation in type 2 diabetes: a systematic review with meta-analysis of 16 randomized controlled trials

**DOI:** 10.1186/s12933-020-01105-5

**Published:** 2020-08-26

**Authors:** Wen-jie Li, Xing-qing Chen, Ling-ling Xu, Yuan-qing Li, Bi-hui Luo

**Affiliations:** 1 Nanshan School, Guangzhou Medical University, Jingxiu Road, Panyu District, Guangzhou, Guangdong China; 2 Department of Cardiology, The First Affiliated Hospital of Guangzhou Medical University, 151 Yan Jiang Xi Road, Guangzhou District, Guangdong, China

**Keywords:** Sodium-glucose cotransporter 2 inhibitors, Atrial fibrillation, Atrial flutter, Type 2 diabetes, Meta-analysis

## Abstract

**Background:**

Type 2 diabetes is closely related to an increased risk of atrial fibrillation (AF) and atrial flutter (AFL). Whether sodium-glucose cotransporter 2 (SGLT2) inhibitors can attenuate AF/AFL progression remains unclear.

**Methods:**

We searched electronic databases (PubMed, Embase and ClinicalTrials.gov) from their inception to January 2020 for trials evaluating the AF outcomes of SGLT2 inhibitors in patients with type 2 diabetes. The data search and extraction were conducted with a standardized data form and any conflicts were resolved by consensus. Relative risks (RRs) with 95% confidence intervals (CIs) were used for binary variables, and the weighed mean differences (WMDs) with the standard deviation (SDs) were applied for continuous variables.

**Results:**

We included data from 16 identified trials consisting of 38,335 patients with type 2 diabetes. Incorporated data demonstrated that compared to placebo, SGLT2 inhibitors significantly reduced AF/AFL (RR: 0.76; 95% CI 0.65–0.90; p = 0.001) and all-cause mortality (RR: 0.91; 95% CI 0.83–0.99; p = 0.03). AF/AFL reductions were not modified by age, body weight, glycated haemoglobin (HbA1c), or systolic blood pressure (SBP) at baseline (all p-interactions > 0.3). SGLT2 inhibitors also significantly reduced heart failure events (RR: 0.73; 95% CI 0.64–0.84; p < 0.00001), HbA1c (WMD: − 0.62%; 95% CI − 0.89 to − 0.34; p < 0.00001), body weight (WMD: − 2.12 kg; 95% CI − 2.91 to − 1.34; p < 0.00001), SBP (WMD: − 3.34 mmHg; 95% CI − 4.12 to − 2.56; p < 0.00001), and diastolic blood pressure (DBP) (WMD: − 1.11 mmHg; 95% CI − 1.62 to − 0.60; p < 0.0001). Of note, cerebrovascular events and myocardial infarction did not increase in patients taking SGLT2 inhibitors.

**Conclusion:**

SGLT2 inhibitors may confer a specific AF/AFL-reduction benefit in the susceptible type 2 diabetes population, regardless of age, body weight, HbA1c, and systolic blood pressure at baseline. Such an AF/AFL-reduction benefit may be partly attributed to pharmacological effects on reductions in HbA1c, body weight, blood pressure, and the occurrence of heart failure.

## Introduction

Atrial fibrillation (AF) and atrial flutter (AFL) are leading causes of mortality worldwide that frequently result in cerebrovascular events [1]. It is generally acknowledged that type 2 diabetes is significantly associated with an increased risk of developing AF/AFL [2, 3]. The underlying mechanisms can be attributed to insulin resistance that engenders myocardial remodelling, or expansion of epicardial adipose tissue which leads to inflammation-related cardiac fibrosis and the change of atrial electrical properties [4–6]. However, whether hypoglycaemic agents alter the risk of AF/AFL is incompletely understood [3].

Sodium-glucose cotransporter 2 inhibitors (SGLT2i), a novel class of oral hypoglycaemic medication, have been demonstrated to potentially reduce the risk of cardiovascular outcomes, especially heart failure (HF) and all-cause mortality, in several large placebo-controlled randomized controlled trials (RCTs) and meta-analyses [7–10]. The key mechanisms may be explained by the potentially direct myocardial effects of SGLT2 inhibitors on diuresis and myocardial metabolism [11]. Furthermore, HF and AF/AFL are closely aligned and share risk factors such as diabetes, obesity and hypertension [12, 13]. Atrial structural and neurohormonal alterations in HF are extremely likely to promote the development and progression of AF/AFL [12]. Thus, we hypothesize that at the same time, pharmacologic therapies for HF could contribute to the reduced AF/AFL risk. Additionally, SGLT2 inhibitors have been reported to decrease glycated haemoglobin (HbA1c), body weight and blood pressure [14]. Such findings also imply crucial roles of SGLT2 inhibitors in AF/AFL improvement. Nonetheless, no RCTs to date, other than a post hoc analysis of the DECLARE-TIMI 58 trial [15] address the relationship between SGLT2 inhibitors and the risk of AF/AFL in the population. Hence, the overarching purpose of the present meta-analysis was to pool data from all placebo-controlled RCTs that evaluated AF/AFL outcomes of SGLT2 inhibitors, from which we gained more reliable assessments of the efficacy and safety of specific results overall and in relevant subgroups.

## Methods

Data sources and search strategy, data acquisition, inclusion and exclusion criteria, outcome measurements, quality assessment and statistical methods in the present report were performed in accord with the Preferred Reporting Items for Systematic Reviews and Meta-analysis (PRISRM) guidelines [16].

### Data sources and search strategy

An article search was carried out in January 2020 without restrictions on publication types, regions, sample sizes or languages. The main data sources were PubMed, Embase, and ClinicalTrials.gov. We screened unpublished and published RCTs through MeSH terms and their combinations related to AF with a free text search for SGLT2 inhibitors from inception of the above electronic databases. The detailed search algorithm is presented Additional file 1. References listed in identified studies and function of the related articles were also assessed to broaden the scope of search. When there were multiple RCTs with the same patient cohorts, the most recently published trial was included.

### Inclusion and exclusion criteria

A pair of 2 independent reviewers (WJL and XQC) identified the titles, abstracts and full-texts of all citations. Independent reviewers (WJL and XQC, LLX and YQL), again working in pairs, identified the full-text version of qualified references. Any disagreement was resolved by the third senior author (BHL).

We included studies if: they were RCTs published in English, enrolled patients aged 18 years or older who had type 2 diabetes mellitus, were performed in out- or inpatient-settings, compared SGLT2 inhibitors with placebo, and reported outcomes of interest. We excluded those focused on patients with type 1 diabetes mellitus or malignant tumours. Letters to the editor, editorials, case reports, review articles, and animal model literature were eliminated.

### Outcomes of interest

The pre-set overarching outcome of interest was the incidence of AF/AFL (the composite of new-onset and recurrent AF/AFL). We also examined all-cause mortality, HF, cerebrovascular events, and myocardial infarction as the primary outcomes. The secondary outcomes were other safety endpoints, including urinary tract infections, and the effects of SGLT2 inhibitors on changes in HbA1c, body weight loss, systolic blood pressure (SBP) and diastolic blood pressure (DBP). Cerebrovascular events were defined as the combination of cerebral haemorrhage and ischaemic stroke.

Prespecified subgroup analyses were conducted to compare the SGLT2 inhibitor treatment effect between different agent types, the proportion of subjects by sex or duration of treatments. We predefined high doses of 10 mg for dapagliflozin, 300 mg for canagliflozin, and 25 mg for empagliflozin and low doses of 2.5 mg or 5 mg for dapagliflozin, 100 mg for canagliflozin, and 10 mg for empagliflozin. Subgroups of interest were also present in AF/AFL, including age (< 60 vs. ≥ 60 years), body weight (< 90 vs. ≥ 90 kg), HbA1c (< 8.1 vs. ≥ 8.1%), and SBP (< 135 vs. ≥ 135 mmHg).

### Data extraction and quality assessment

Using a standardized form, 2 independent reviewers (WJL and XQC) manually extracted information from the included studies as follows: (1) study design, patient characteristics, follow-up durations; (2) comparisons, interventions, background intervention, outcomes at different time points or with different agent types; and (3) related items for outcomes of interest. We also tried to contact authors of the trials screened in our search by email in order to obtain additional data where necessary. Any missing data were found in ClinicalTrials.gov for RCTs.

The Cochrane risk-of-bias tool was applied to assess the methodological quality of the RCTs [17]. Two reviewers (WJL and XQC) independently assessed the risk of bias of the included studies at the study, intervention and outcome levels. Risk-of-bias assessments with disagreement were reanalysed and discussed until a consensus was reached.

### Grading the strength of evidence

We evaluated the applicability of the analysis outcomes via the Agency for Healthcare Research and Quality (AHRQ) criteria by using the Strength of Evidence (SOE). Two reviewers (WJL and LLX) independently graded the SOE for each of the outcomes of interest as low, moderate or high. Any conflict was resolved by consulting a third reviewer (BHL).

### Statistical analysis

All meta-analyses were performed using RevMan 5.3 and Stata 14.0. We used pooled relative risks (RRs) with corresponding 95% confidence intervals (CIs) for the incidence of AF/AFL and predefined safety endpoints in patients with type 2 diabetes mellitus who received standard treatment with or without SGLT2 inhibitors. Weighed mean differences (WMDs) with standard deviation (SD) were applied for continuous variables.

To assess the extent to which the outcomes of the included studies are consistent. Heterogeneity was assessed by using the Cochrane Q test and Higgins and Thompsons’ I^2^ before the meta-analysis. For the Q test, we determined that a threshold *p* value < 0.1 was statistically significant. Additionally, heterogeneity was deemed to be low if I^2^ was < 50%; otherwise, it was high if I^2^ was > 50% [17]. If high heterogeneity between studies was found, we used the random-effects (RE) model; otherwise, the fixed-effects (FE) model was applied. We further investigated heterogeneity among studies by conducting subgroup analyses, meta-regression and sensitivity analyses. Interaction terms were used to evaluate whether the occurrence of AF/AFL would change with different factors across subgroups throughout Revman 5.3 software. A p-value < 0.05 was set as significant factors that were associated with the occurrence of AF/AFL. Publication bias was investigated by the use of funnel plots and Egger’s test. P-values < 0.05 (two-sided) were considered statistically significant and we did not adjust for multiple testing.

To investigate heterogeneity, univariable meta-regression was carried out if more than 10 trials were included in the meta-analysis. We considered a p-value less than 0.05 to be statistically significant. The Monte Carlo permutation test (5001 permutations, p < 0.1) was subsequently used to calculate p values and decrease the false-positive/negative findings in the meta-regression. The following covariates were investigated: sample size; follow-up of trials, and proportion of females (%). A P value less than 0.1 was set as the criterion for heterogeneity source. Finally, sensitivity analyses were performed to assess the robustness of the outcomes by removing each included study individually to explore the remaining overall estimates of AF/AFL events.

## Results

**Fig. 1:**
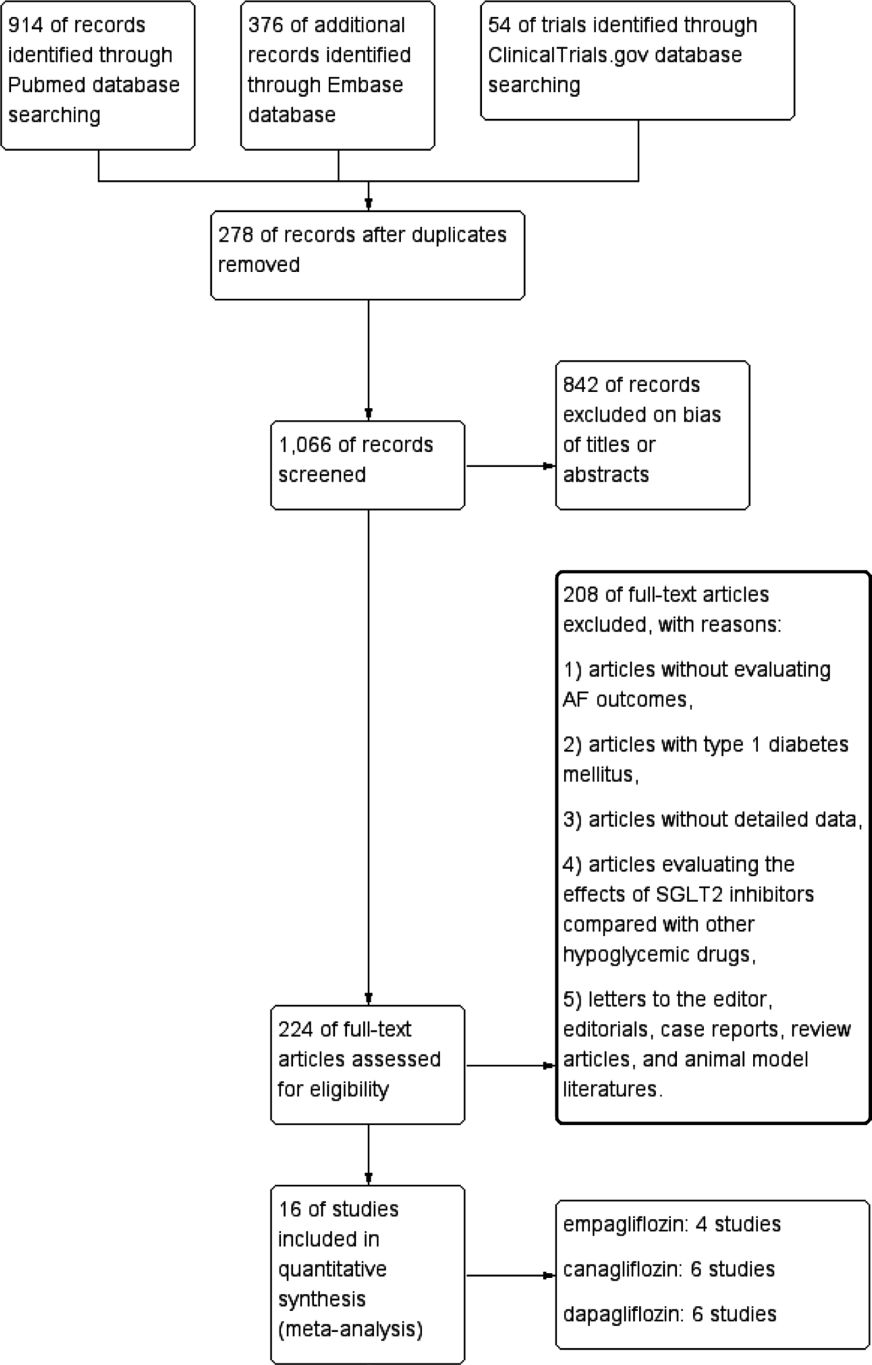
Flow diagram of articles identified, included, and excluded

Sixteen RCTs [7, 8, 18–31] including 38,335 patients with type 2 diabetes mellitus (20,914 patients who received SGLT2 inhibitors and 17,421 patients with placebo) matched our predefined inclusion criteria and were included in the final meta-analysis (Fig. 1). All publications were full-text articles. The references listed in the included studies and related articles did not provide additional studies for further analysis. Agreement between the two independent reviewers was 94% for study selection, 93% for quality assessment of included trials and 91% for the SOE.

### Characteristics of eligible studies

The characteristics of the included studies are clearly illustrated in Table 1. The studies were published from 2012 to 2019 and had sample sizes ranging from 269 patients to 17,160 patients. The proportion of females ranged from 33.1% to 54.4%. Among the included studies, 12 trials [8, 18–20, 22–25, 27, 28, 30, 31] evaluated the effects of different pharmacologic dosages. For the study regarding pharmacologic intervention, four articles [18, 25, 27, 31] included empagliflozin, six trials [7, 8, 19, 23, 24, 30] used canagliflozin, and six studies [20–22, 26, 28, 29] included dapagliflozin therapy. Across all sixteen studies, the median follow-up duration was 1.8 years.

**Table 1:** Characteristics of included RCTs

Study (trial name)	Study design (NCT number)	Mean age (SD)		Health conditions	Number of patients			Interventions	Background hypoglycaemic therapy	Mean follow-up (weeks)
		Treatment	Control		Treatment (Female/male)	Control (Female/male)	Female (%)			
Dapagliflozin										
Wilding et al. [22]	RCT (NCT00673231)	59.5 ± 8.1	58.8 ± 8.6	Type 2 diabetes mellitus	607 (320/287)	193 (98/95)	52.2	Dapagliflozin (2.5/5/10 mg) Matching placebo	Insulin	48
Bailey et al. [28]	RCT (NCT00528879)	53.7 (NA)	54.0 (NA)	Type 2 diabetes mellitus	409 (194/215)	137 (62/75)	46.9	Dapagliflozin (2.5/5/10 mg) Matching placebo	Metformin	102
Leiter et al. [21]	RCT (NCT01042977)	63.9 ± 7.6	63.6 ± 7.0	Type 2 diabetes mellitus Cardiovascular disease	480 (159/321)	482 (159/323)	33.1	Dapagliflozin (10 mg) Matching placebo	Insulin	24
Mathieu et al. [29]	RCT (NCT016 6320)	55.2 ± 8.6	55.0 ± 9.6	Type 2 diabetes mellitus	160 (90/70)	160 (84/76)	54.4	Dapagliflozin (10 mg) Matching placebo	Metformin saxagliptin	24
NCT00528372 (2015)	RCT (NCT00528372)	NA	NA	Type 2 diabetes mellitus	410 (212/198)	75 (44/31)	52.7	Dapagliflozin (2.5/5/10 mg) Matching placebo	Metformin	102
NCT01730534 (2019) DARELARE-TIMI58	RCT (NCT01730534)	63.9 ± 6.8	64.0 ± 6.8	Type 2 diabetes mellitus Cardiovascular disease	8582 (3171/5411)	8578 (3251/5327)	37.4	Dapagliflozin (10 mg) Matching placebo	Metformin Insulin Sulfonylurea DPP-4i GLP-1 receptor agonist	202
Canagliflozin										
Wilding et al. [23] CANTATA-MSU Trial	RCT (NCT01106625)	56.8 ± 9.7	56.7 ± 8.3	Type 2 diabetes mellitus	313 (150/163)	156 (80/76)	49	Canagliflozin (100/300 mg) matching placebo	Metformin Suiphonylurea	52
Yale et al. [24]	RCT (NCT01064414)	68.7 ± 8.2	68.2 ± 8.4	Type 2 diabetes mellitus Chronic kidney disease	179 (73/106)	90 (33/57)	36.1	Canagliflozin (100/300 mg) Matching placebo	Insulin Sulphonylurea	52
Bode et al. [30]	RCT (NCT01106651)	63.9 ± 6.2	63.2 ± 6.2	Type 2 diabetes mellitus	477 (224/253)	237 (94/143)	44.5	Canagliflozin (100/300 mg) Matching placebo	Insulin Sulphonylurea	104
NCT01989754 (2018) CANVAS-R Trial	RCT (NCT01989754)	63.9 ± 8.42	64 ± 8.28	Type 2 diabetes mellitus Chronic kidney disease	2904 (1111/1794)	2903 (1053/1854)	37.3	Canagliflozin (100/300 mg) Matching placebo	Metformin Insulin Sulfonylurea DPP-4i GLP-1 receptor agonist	187
NCT01032629 (2018) CANVAS Trial	RCT (NCT01032629)	62.5 ± 8.1	62.3 ± 7.9	Type 2 diabetes mellitus Cardiovascular Diseases	2888(983/1905)	1442(486/956)	33.9	Canagliflozin (100/300 mg) Matching placebo	Metformin Insulin Sulfonylurea DPP-4i GLP-1 receptor agonist	202
Perkovic et al. [7] CREDENCE Trial	RCT (NCT02065791)	62.9 ± 9.2	63.2 ± 9.2	Type 2 diabetes mellitus Chronic kidney disease	2202(762/1440)	2199(732/1467)	33.9	Canagliflozin (100 mg) Matching placebo	Metformin Insulin Sulfonylurea DPP-4i GLP-1 receptor agonist	125
Empagliflozin										
Kovacs et al. [27]	RCT (NCT01210001)	54.5 ± 9.4	54.6 ± 10.5	Type 2 diabetes mellitus	333 (165/168)	165(92/73)	51.6	Empagliflozin (10/25 mg) Matching placebo	Metformin Pioglitazone	24
Barnett et al. [31]	RCT (NCT01164501)	63.7 ± 8.9	64.1 ± 8.7	Type 2 diabetes mellitus Chronic kidney disease	419 (170/249)	319 (138/181)	41.7	Empagliflozin (10/25 mg) Matching placebo	Metformin Pioglitazone Insulin	52
Rosenstock et al. [25]	RCT (NCT01011868)	59.2 (NA)	58.1(NA)	Type 2 diabetes mellitus	324 (138/186)	170 (80/90)	42.4	Empagliflozin (10/25 mg) Matching placebo	Insulin	78
NCT01734785 (2016)	RCT (NCT01734785)	54.9 ± 9.7	55.9 ± 9.6	Type 2 diabetes mellitus	222 (85/137)	110 (49/61)	40.4	Empagliflozin (10/25 mg) Matching placebo	Metformin	24

*DPP-4i* dipeptidyl peptidase-4 inhibitor, *GLP-1* glucagon-like peptide-1, *NA* not available

**Fig. 2:**
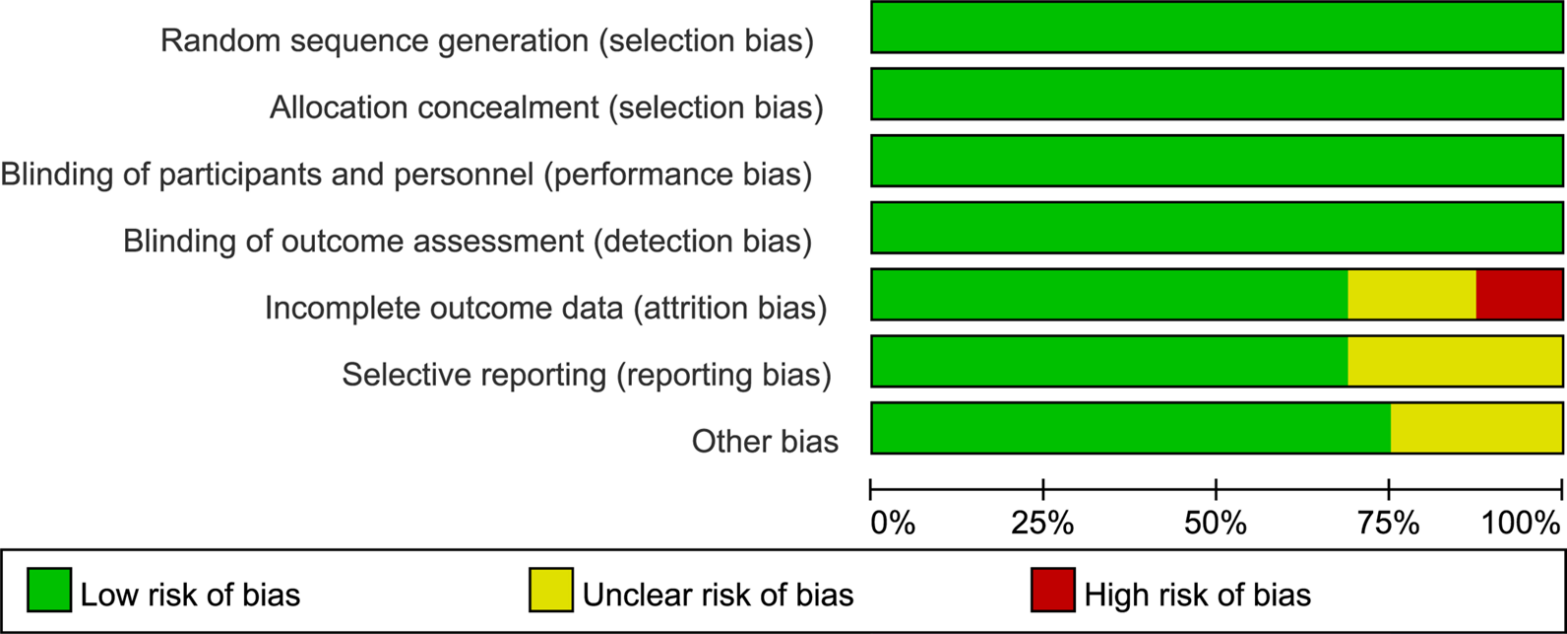
Methodological quality assessment of included randomized controlled trials

Quality assessment items are presented in Fig. 2. In 16 trials, most studies were of considerably high methodological quality, indicating minimal selection bias or implementation bias. All data were considerably complete and bias from the blinding method did not appear in any of the included studies.

### Results of meta-analysis

The summarized outcomes of our meta-analysis are shown in Table 2. The sources for data extraction are indicated in Additional file 1. Forest plots, demonstrating the effect size of each analysed study, are presented in Figs. 3, 4 and Additional file 1: Figures S1–S13.

**Table 2:** Results of meta-analysis comparison of SGLT2 inhibitors and placebo

Outcomes of interest	Numbers of analyzed studies	SGLT2i patients	Placebo patients	RR (95% CI)	p value	Study heterogeneity		
						χ^2^	I^2^, %	p value
Primary outcomes								
Incidence of AF/AFL	16	20,914	17,421	0.76 (0.65 to 0.90)	0.001*	7.72	0	0.93
All-cause mortality	12	19,809	16,920	0.91 (0.83 to 0.99)	0.03*	4.06	0	0.97
Heart failure	10	18,701	16,485	0.73 (0.64 to 0.84)	< 0.00001*	10.43	14	0.32
Cerebrovascular events	13	20,199	16,986	1.06 (0.85 to 1.32)	0.19	15.99	25	0.19
Myocardial infarction	13	19,747	16,949	0.95 (0.78 to 1.16)	0.65	6.81	0	0.87
Secondary outcomes								
Urinary tract infection rate	15	12,332	8842	1.17 (1.03 to 1.32)	0.01*	14.4	3	0.42
Adjusted mean HbA1c (%) change from baseline								
Low dosage	9	2652	2601	− 0.62 (− 0.89 to − 0.34)	< 0.00001*	173.58	95	< 0.00001*
High dosage	11	3214	3230	− 0.70 (− 0.91 to − 0.50)	< 0.00001*	166.91	94	< 0.00001*
Adjusted mean body weight loss (kg) change from baseline								
Low dosage	5	778	776	− 2.12 (− 2.91 to − 1.34)	< 0.00001*	21.08	81	0.0003*
High dosage	7	1398	1405	− 1.89 (− 2.13 to − 1.65)	< 0.00001*	8.33	28	0.21
Adjusted mean blood pressure (mm Hg) change from baseline								
Systolic blood pressure								
Low dosage	6	2283	2257	− 3.34 (− 4.12 to − 2.56)	< 0.00001*	4.18	0	0.52
High dosage	7	2709	2736	− 4.11 (− 4.86 to − 3.36)	< 0.00001*	11.51	48	0.07
Diastolic blood pressure								
Low dosage	6	2283	2257	− 1.11 (− 1.62 to − 0.6)	< 0.0001*	4.37	0	0.50
High dosage	6	2236	2257	− 1.69 (− 2.17 to − 1.12)	< 0.0001*	4.77	0	0.44

*SGLT2* sodium-glucose co-transporter 2, *AF* atrial fibrillation, *AFL* atrial flutter, *RR* relative risk, *CI* confidence interval

**Fig. 3:**
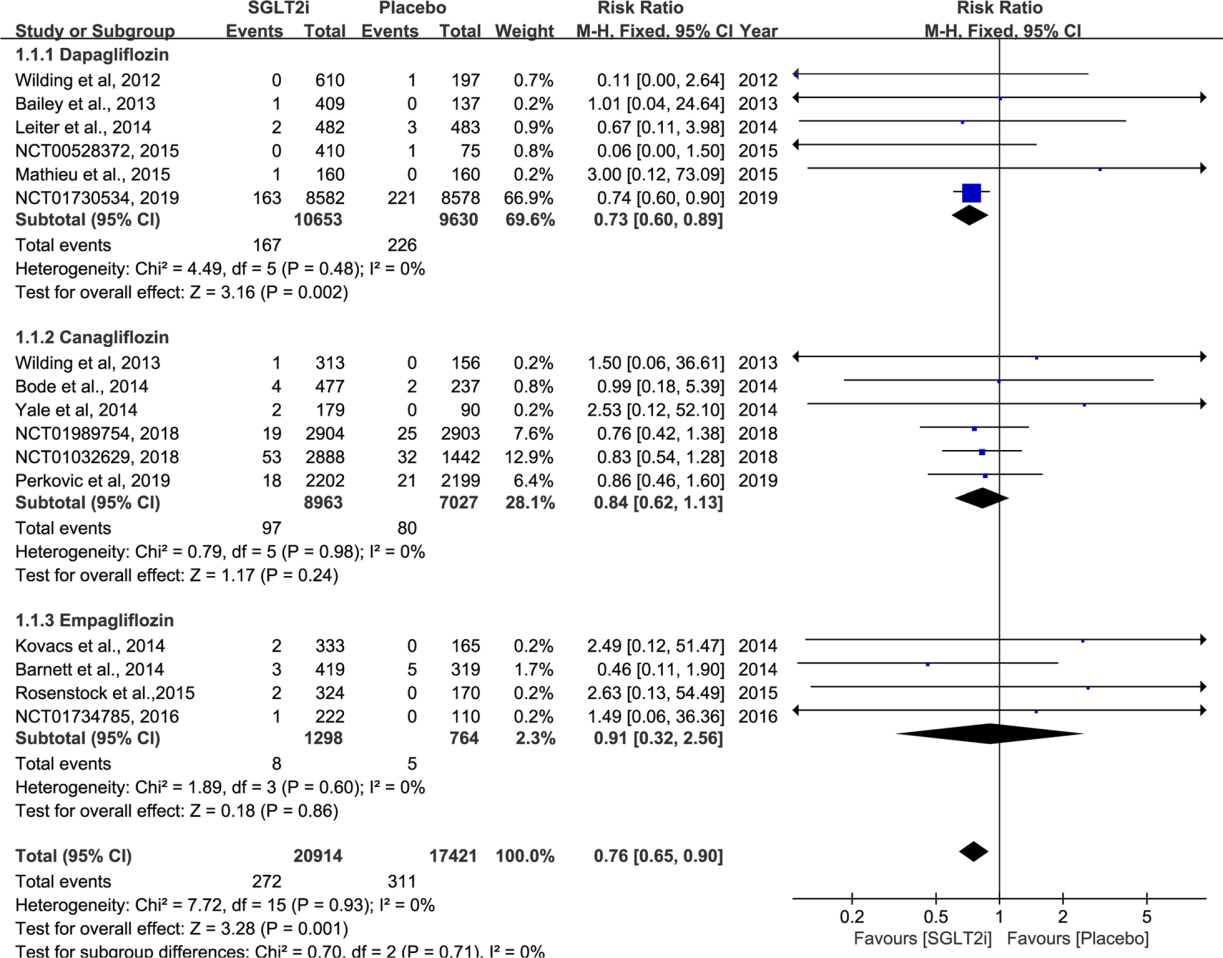
Forest plot and meta-analysis of atrial fibrillation/atrial flutter events. Weights are from the fixed-effect analysis. The solid line across the square represents the 95% confidence interval (CI)

### Primary outcomes

#### Incidence of AF/AFL

Pooling 16 studies assessing the incidence of AF/AFL (SGLT2 inhibitors, 20,914 patients; placebo, 17,421 patients) showed that SGLT2 inhibitors significantly decreased the incidence of reported AF/AFL events compared with placebo (RR: 0.76; 95% CI 0.65–0.90; p = 0.001; high SOE). The pooled trials without background hypoglycaemic therapy also demonstrated a significant difference (RR: 0.76; 95% CI 0.65–0.90; p = 0.001). In subgroups of different agent types, dapagliflozin was associated with significantly reduced AF/AFL (RR: 0.73; 95% CI 0.60–0.89; p = 0.02), while canagliflozin and empagliflozin showed no evident effect of reducing AF/AFL events (RR_canagliflozin_: 0.84; 95% CI 0.62–1.13; p = 0.24; RR_empagliflozin_: 0.91; 95% CI 0.32–2.56; p = 0.6) (Fig. 3).

SGLT2 inhibitor treatment was associated with a significantly reduced AF/AFL incidence, when analysing trials with follow-ups longer 2 years (RR: 0.76; 95% CI 0.64–0.89; p = 0.001), whereas no significant differences were observed with follow-up durations shorter 2 years (RR: 0.91; 95% CI 0.44–1.89; p = 0.79) (Additional file 1: Figure S1). Moreover, interaction tests demonstrated p values were greater than 0.05, suggesting that the occurrence of AF/AFL was not modified by age, body weight, HbA1c, or systolic blood pressure (Table 2).

#### All-cause mortality

**Fig. 4:**
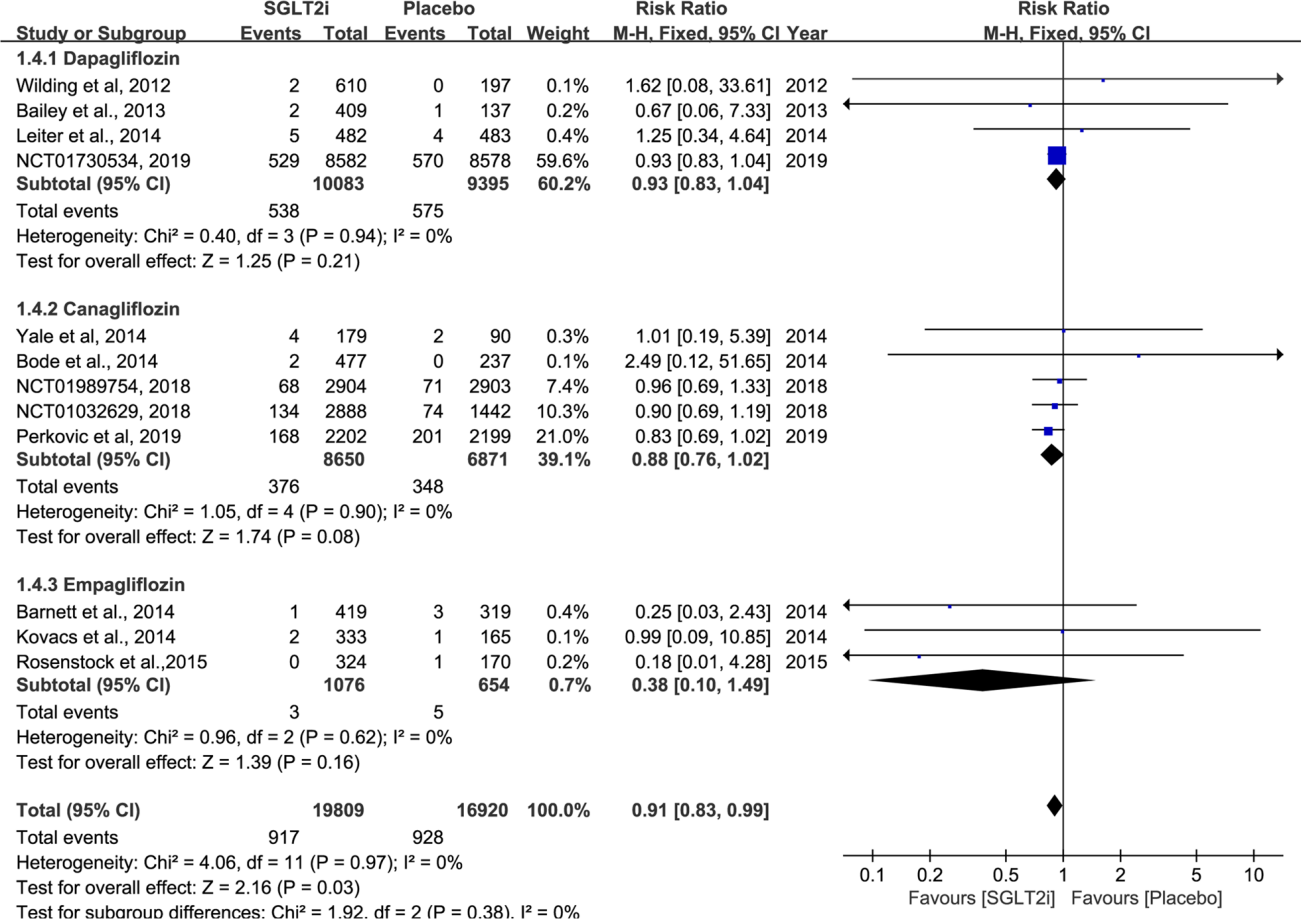
Forest plot and meta-analysis of all-cause mortality. Weights are from the fixed-effect analysis. The solid line across the square represents the 95% confidence interval (CI)

Apart from 4 studies [18, 20, 23, 29], 12 studies reported all-cause mortality (SGLT2 inhibitors, 19,809 patients, 917 events; placebo, 16,920 patients, 928 events). The use of SGLT2 inhibitors significantly reduced all-cause mortality (RR: 0.91; 95% CI 0.83–0.99; p = 0.03; moderate SOE) (Fig. 4). In view of the closed association among age, diabetes mellitus and all-cause mortality, we eliminated the two studies with the oldest populations [8, 24]. SGLT2 inhibitor treatment was more likely to be associated with lower all-cause mortality after adjustment (RR_adjusted_: 0.90; 95% CI 0.82–0.99; p = 0.03; moderate SOE). Moreover, on further subgroup analysis for follow-ups, reduced all-cause mortality was associated with long follow-up durations (RR_>2 years_: 0.91; 95% CI 0.83–0.99; p = 0.03), whereas no significant difference was observed in trials with short follow-up durations (RR_<2 years_: 0.81; 95% CI 0.38–1.74; p = 0.59).

#### Heart failure

Ten studies [7, 8, 19–21, 24, 26, 30, 31] including 35,186 patients evaluated heart failure (SGLT2 inhibitors, 18,701 patients, 356 events; placebo, 16,485 patients, 459 events). The aggregated data showed a significantly lower rate of heart failure in patients treated with SGLT2 inhibitors than in those treated with placebo (RR: 0.73; 95% CI 0.64–0.84; p < 0.00001; high SOE). Significant reductions were noted in patients treated with dapagliflozin (RR: 0.79; 95% CI 0.66–0.95; p = 0.01) and canagliflozin (RR: 0.66; 95% CI 0.53–0.82; p = 0.0001). However, no difference was seen in the empagliflozin group (RR: 0.11; 95% CI 0.01–2.10; p = 0.14) (Additional file 1: Figure S2). Given the potential association between AF/AFL and HF [12], we subsequently performed a meta-regression to investigate their correlation.

#### Cerebrovascular events

Out of the 17 selected studies, 13 studies [7, 8, 19–23, 25, 26, 28, 30, 31] evaluated cerebrovascular events (SGLT2 inhibitors, 20,199 patients, 180 events; placebo, 16,986 patients, 150 events). The analysis showed that cerebrovascular events did not differ between groups (RR: 1.06; 95% CI 0.85–1.32; p = 0.59; moderate SOE). Consistent results were shown in the subgroup analysis (RR _dapagliflozin_: 1.25; 95% CI 0.96–1.63; p = 0.09; RR _canagliflozin_: 0.70; 95% CI 0.47–1.06; p = 0.09; RR _empagliflozin_: 1.93; 95% CI 0.30–12.92; p = 0.49) (Additional file 1: Figure S3).

#### Myocardial infarction

Apart from 3 trials [18, 22, 27], thirteen studies evaluated the occurrence of myocardial infarction (SGLT2 inhibitors, 19,747 patients, 200 events; placebo, 16,949 patients, 186 events). SGLT2 inhibitors did not increase the risk of myocardial infarction (RR: 0.95; 95% CI 0.78–1.16; p = 0.65; moderate SOE), which was consistent with SGLT2 inhibitor subtypes (RR_dapagliflozin_: 0.95; 95% CI 0.74–1.23; p = 0.71; RR_canagliflozin_: 0.97; 95% CI 0.71–1.33; p = 0.87; RR_empagliflozin_: 0.62; 95% CI 0.11–3.37; p = 0.58) (Additional file 1: Figure S4).

### Secondary outcomes

#### Urinary tract infection rate

Apart from 1 trial [26], fifteen studies including 21,174 patients evaluated the urinary tract infection rate (SGLT2 inhibitors, 12,332 patients, 657 events; placebo, 8842 patients, 395 events). The pooled evidence showed significantly lower urinary tract infection rate in the placebo group than in the SGLT2i group (RR: 1.17; 95% CI 1.03–1.32; p = 0.01; moderate SOE). When divided into different SGLT2i interventions, dapagliflozin significantly increased the risk of urinary tract infection (RR_dapagliflozin_: 1.56; 95% CI 1.15–2.11; p = 0.004), while no significant differences were found for canagliflozin (RR_canagliflozin_: 1.09; 95% CI 0.93–1.28; p = 0.27) or empagliflozin (RR_empagliflozin_: 1.11; 95% CI 0.86–1.45; p = 0.42) (Additional file 1: Figure S5).

#### Adjusted mean HbA1c (%) change from baseline

In total, 11 trials [18, 19, 21–25, 27–30] including 9734 patients investigated adjusted mean HbA1c (%) change from baseline with detailed 95% CIs or standard errors (SEs). SGLT2 inhibitors could reduce the levels of HbA1c (ranging from 0.26% to 1.9%). The pooled data showed significant differences in the low- and high-dosage groups (low dosage: WMD: − 0.62%; 95% CI − 0.89 to − 0.34; p < 0.00001; high dosage: WMD: − 0.70%; 95% CI − 0.91 to − 0.50; p < 0.00001; moderate SOE) but with significant heterogeneity (low dosage: I^2^ = 95%, p < 0.00001; high dosage: I^2^ = 94%, p < 0.00001) (Additional file 1: Figures S6 and S7). Sensitivity analysis, meta-regression and Monte Carlo permutation tests were conducted to investigate the main sources of heterogeneity.

#### Adjusted mean body weight loss (kg) change from baseline

Pooling the data from the 7 trials [18, 20–22, 25, 27, 28] with detailed 95% CIs or SEs assessing mean body weight loss changes from baseline showed that SGLT2 inhibitors significantly reduced body weight (ranging 0.98 kg to 3.06 kg; low dosage: WMD: -2.12 kg; 95% CI − 2.91 to − 1.34; p < 0.00001; high dosage: WMD: − 1.94 kg; 95% CI − 2.18 to − 1.69; p < 0.00001; moderate SOE) (Additional file 1: Figures S8 and S9).

#### Adjusted blood pressure (mm Hg) change from baseline

Seven trials [19, 21, 23, 25, 27, 28, 30] with detailed 95% CIs or SEs reported SBP changes from baseline, and 6 trials [19, 23, 25, 27, 28, 30] reported DBP. Incorporating the data showed that decreased blood pressure was significantly associated with SGLT2 inhibitors for SBP (ranging 0.7 mm Hg to 6.49 mm Hg; low dosage: WMD: − 3.34 mm Hg; 95% CI − 4.12 to − 2.56; p < 0.00001; high dosage: WMD: − 4.11 mm Hg; 95% CI − 4.86 to − 3.36; p < 0.00001; moderate SOE) and DBP (ranging 0.1 mm Hg to 4.51 mm Hg; low dosage: WMD: − 1.11 mm Hg; 95% CI − 1.62 to − 0.60; p < 0.0001; high dosage: WMD: -1.69 mm Hg; 95% CI − 2.17 to − 1.21; p < 0.00001; moderate SOE) (Additional file 1: Figures S10, S11, S12, S13).

#### Sensitivity analysis and major sources of heterogeneity

All analysed results presented relatively low heterogeneity except for HbA1c changes from baseline (low dosage: I^2^ = 95%, p < 0.00001; high dosage: I^2^ = 94%, p < 0.00001) (Table 2). As a result, the sensitivity analysis for HbA1c change demonstrated that NCT01734785(2016) [18] and NCT01032629(2018) [19] seemed to be different from the rest of the trials. The exclusion of these studies resulted in heterogeneity changes for low dosage (I^2^ = 29%, p = 0.19), indicating that these included trials may be the sources of heterogeneity. There was no evident change for high dosage (I^2^ = 72%, p = 0.002). Thus, the meta-regression and Monte Carlo permutation test were subsequently conducted to further investigate the sources of heterogeneity.

#### Meta-regression and Monte Carlo permutation test

Meta-regression was used to investigate the heterogeneity source of included studies that evaluated HbA1c change from baseline for high dosage SGLT2 inhibitors. The results showed that the proportion of female subjects (coefficient = 0.0027; p = 0.874), follow-up (coefficient = 0.0029; p = 0.181), and sample size (coefficient = 0.00022; p = 0.171) were not sources of heterogeneity (Additional file 1: Figure S14). A total of 5001 iterations were run in the permutation test to reduce the chance of a false positive of p values for female proportion (adjusted p = 0.891), follow-up (adjusted p = 0.149), and sample size (adjusted p = 0.092), indicating that the sample size may be the identified source of heterogeneity. In addition, meta-regression showed no significant correlation between AF/AFL and HF (coefficient = 0.034; p = 0.751), and Monte Carlo permutation test showed an adjusted p = 0.559.

#### Publication bias

**Fig. 5:**
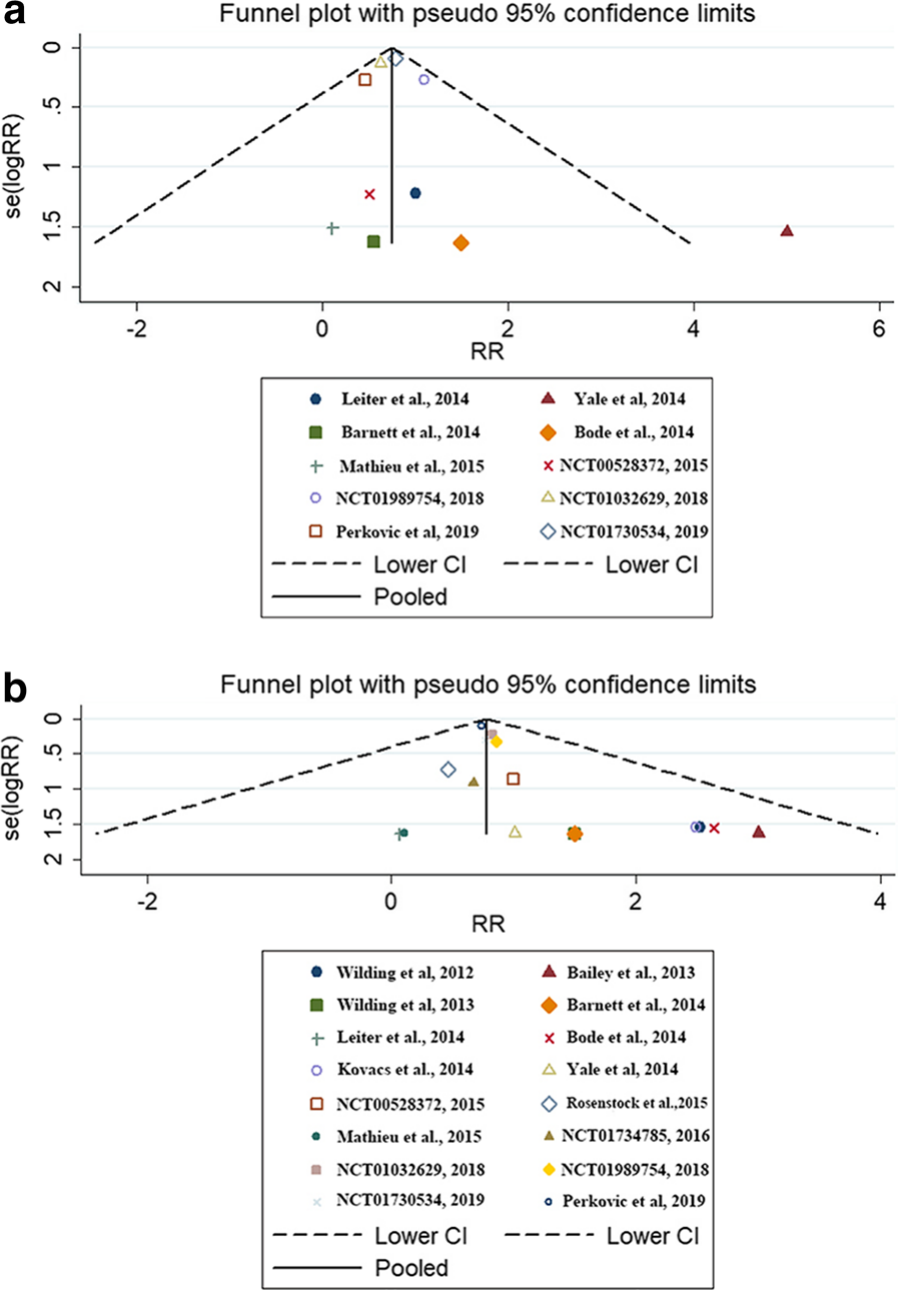
Funnel plots illustrating meta-analysis: **a** heart failure and **b** atrial fibrillation/atrial flutter

Funnel plots were constructed to resolve the publication bias for the studies evaluating HF (Egger’s test p = 0.936, 95% CI − 1.152 to 1.072) and AF/AFL (Egger’s test p = 0.568, 95% CI − 0.365 to 0.640). Figure 5 shows the trials included in this meta-analysis that reported HF and AF/AFL. Overall, the scatter points were dispersed symmetrically in the funnel plot. All the evidence suggests that the probability of publication bias is low.

## Discussion

To the best of our knowledge, this is the first attempt to systematically evaluate the relationship between SGLT2 inhibitors and AF/AFL in type 2 diabetes, and the totality of the present findings emphasises several patterns. First, SGLT2 inhibitors demonstrated great benefits in reducing the relative risk of AF/AFL in type 2 diabetes. This effect was consistent regardless of age, HbA1c, blood pressure and body weight and was more obvious with a long duration of SGLT2 inhibitor treatment. Second, for the specific results the clinical effects of SGLT2 inhibitors depended on the patient population to which they are applied. The reduction in all-cause mortality was apparent in patients treated with SGLT2 inhibitors, especially in the younger population. Third, the use of SGLT2 inhibitors did not induce an extra risk of cerebrovascular events and myocardial infarction. This is especially remarkable because no individual trial has evaluated these findings, and it was revealed only after analysing the results. Largely, there were no obvious heterogeneity in the above analyses from the results of heterogeneity tests, suggesting the reliability of the present outcomes.

The present meta-analysis outlined that SGLT2 inhibitors had great benefit in reducing the risk of AF/AFL. Notably, the reduction in AF/AFL was strongly supported by the recent large-scale trials, NCT01730534 [26] and NCT01032629 [19], whose results were different from those of other early trials. The inconsistent results may be derived from the small sample sizes and short follow-up periods of previous studies, which might limit the ability to identify AF/AFL-reduction effects and lead to false-negative findings. Despite extensive exploratory studies, the mechanisms of action of SGLT2 inhibitors in AF/AFL treatment remain unclear. The present incorporated data demonstrated favourable effects on reducing HbA1c, blood pressure and body weight in patients randomized to SGLT2 inhibitors, which might be of importance in attenuating AF/AFL progression. On the one hand, increased glucose excretion essentially leads to additional osmotic diuresis, which in turn will cause a reduction in arterial blood pressure and retard myocardial structural remodelling followed by atrial fibrosis [32]. On the other hand, glucose lost in the urine with diuresis may contribute to weight loss and be maintained for a long time, thus reducing atrial dilation and the occurrence of AF [33–35]. Many AF/AFL risk factors are potentially reversible [36]. In addition, our further analysis found that AF/AFL reductions were consistent across subgroups including age, HbA1c, blood pressure and body weight. Therefore, natriuresis followed by reduction in blood pressure and body weight induced by SGLT2 inhibitors may confer a specific AF/AFL-reduction benefit in this susceptible population.

Likewise, atrial fibrosis has central roles in pathogenic remodelling in HF [37]. We confirmed that HF events were significantly decreased in patients randomized to SGLT2 inhibitors. Reductions in both HF and AF/AFL and their concomitant interventions and downstream complications might subsequently reduce the risk of all-cause mortality in our present work [3, 38]. In particular, we found that this effect could be enhanced with therapy of a longer duration. The underlying cause may be the negative correlation between the duration of therapy with SGLT2 inhibitors and HbA1c levels [39]. Our further analysis found that all-cause mortality reductions slightly differed between age groups and appeared to be more distinct in younger patients. This finding may be attributed to vulnerability to comorbidities and competing risks from other diseases in the elderly population [40].

Numerous investigations in diabetic animal models have revealed that SGLT2 inhibitors directly target the amelioration of cardiac fibrosis. Shao et al. [41] found that glycemic control with SGLT2 inhibitors notably mitigated atrial remodelling and cardiac fibrosis through the improvement of mitochondrial function. Habibi et al. [42] also discovered that the SGLT2 inhibitor empagliflozin could improve pro-fibrosis signalling and related interstitial fibrosis. Of note, the pharmacological effects on ameliorating cardiac fibrosis caused by AF/AFL appear to be different from those in HF. In our meta regression, no clinical correlation between AF/AFL and HF was observed. This finding implied that the AF/AFL-reduction effects of SGLT2 inhibitors may be partly independent of HF improvement. Similarly, the analysis of the DECLARE-TIMI 58 trial suggested that dapagliflozin could lower the AF/AFL risk in type 2 diabetes patients irrespective of history of HF [15]. More large-scare investigations are required to corroborate this finding.

Overall, SGLT2 inhibitors are well tolerated and generally safe agents. Because of the higher levels of urine glucose caused by the glycosidic effect of SGLT2 inhibitors [43], relatively high prevalence of urinary tract infections was noted in our analysis. Of note, in the present study the effect of dapagliflozin on urinary tract infections was more manifest than that of empagliflozin or canagliflozin. The same conclusion was also drawn in the meta-analysis by Liu et al. [44], who elucidated that dapagliflozin alone is related to a significantly higher risk for urinary tract infections. However, such infections are easy to manage and rarely recur [45]. Additionally, initial concerns on safety signals for cerebrovascular risk were not supported in the present analysis [46]. The drug effect on myocardial infarction is still a topic of meaningful investigation [14].

The data presented herein indicated that SGLT2 inhibitors should be considered in populations with type 2 diabetes for AF/AFL prevention, given that they safely reduced HbA1c, body weight, and blood pressure and widely reduced the risk of HF across the spectrum of these patients. These risk factors did not modify AF/AFL reductions. Moreover, such effects appeared to be more evident with longer durations of SGLT2 inhibitor therapy. Reductions in all-cause mortality could also be expected, which slightly differed in magnitude based on baseline age characteristics, but were present throughout the age range. Patients with diabetes are a particularly susceptible population at increased risk of AF/AFL and HF [3, 47]. A considerable body of large-scale placebo-controlled trials in populations with type 2 diabetes are needed to clarify whether SGLT2 inhibitors exhibit beneficial effects in reducing AF/AFL.

Although this meta-analysis provides the first evidence for a favourable effect of SGLT2 inhibitors on reducing AF/AFL risk, several limitations in our work should be emphasized. First, several small-sized studies did not report AF outcomes. However, the results of our meta-analysis are expected to be statistically stable and robust based on the large sample size. Second, the data that we used lacks information at the individual level, and we failed to identify new and recurrent AF/AFL. Even though we have identified the specific AF/AFL-reduction benefit of SGLT2 inhibitors, further investigations are required to explore the role of SGLT2 inhibitors in both new and recurrent AF/AFL. Third, each patient in the included studies was given background hypoglycaemic therapy, which may influence cardiovascular outcomes to some extent. The results of our work showed that patients with type 2 diabetes had reductions in AF/AFL incidence from the addition of SGLT2 inhibitors to guideline-directed medical therapy.

## Conclusion

Overall, the pleiotropic effects of SGLT2 inhibitors have a great benefit of reducing AF/AFL and all-cause mortality events in a broad type 2 diabetes population, regardless of baseline characteristics including age, HbA1c, systolic blood pressure and body weight.

## Supplementary information

**Additional file 1: Figure S1:** Forest plot and subgroup meta-analysis of atrial fibrillation/atrial flutter events. Weights are from the fixed-effect analysis. The solid line across the square represents the 95% confidence interval (CI). **Figure S2.** Forest plot and meta-analysis of heart failure. Weights are from the fixed-effect analysis. The solid line across the square represents the 95% confidence interval (CI). **Figure S3.** Forest plot and meta-analysis of cerebrovascular events. Weights are from the fixed-effect analysis. The solid line across the square represents the 95% confidence interval (CI). **Figure S4.** Forest plot and meta-analysis of myocardial infarction. Weights are from the fixed-effect analysis. The solid line across the square represents the 95% confidence interval (CI). **Figure S5.** Forest plot and meta-analysis of urinary tract infection rate. Weights are from the fixed-effect analysis. The solid line across the square represents the 95% confidence interval (CI). **Figure S6:** Forest plot and meta-analysis of adjusted mean HbA1c change from baseline for low dosage. Weights are from the random-effect analysis. The solid line across the square represents the 95% confidence interval (CI). **Figure S7.** Forest plot and meta-analysis of adjusted mean HbA1c change from baseline for high dosage. Weights are from the random-effect analysis. The solid line across the square represents the 95% confidence interval (CI). **Figure S8.** Forest plot and meta-analysis of adjusted mean body weight loss change from baseline for low dosage. Weights are from the random-effect analysis. The solid line across the square represents the 95% confidence interval (CI). **Figure S9.** Forest plot and meta-analysis of adjusted mean body weight loss change from baseline for low dosage. Weights are from the random-effect analysis. The solid line across the square represents the 95% confidence interval (CI). **Figure S10.** Forest plot and meta-analysis of adjusted mean SBP change from baseline for high dosage. Weights are from the fixed-effect analysis. The solid line across the square represents the 95% confidence interval (CI). SBP: systolic blood pressure. **Figure S11.** Forest plot and meta-analysis of adjusted SBP change from baseline for high dosage. Weights are from the fixed-effect analysis. The solid line across the square represents the 95% confidence interval (CI). SBP: systolic blood pressure. **Figure S12.** Forest plot and meta-analysis of adjusted DBP change from baseline for low dosage. Weights are from the fixed-effect analysis. The solid line across the square represents the 95% confidence interval (CI). DBP: diastolic blood pressure. **Figure S13.** Forest plot and meta-analysis of adjusted DBP change from baseline for high dosage. Weights are from the fixed-effect analysis. The solid line across the square represents the 95% confidence interval (CI). DBP: diastolic blood pressure. **Figure S14.** Random effect meta-regression for adjusted mean HbA1c (%) change from baseline with the following covariates: (A) female proportion (%) (B) duration of follow-ups (year), and (C) sample sizes.

## Data Availability

Data extracted or analyzed in our work are included in the main text and additional file.

## Abbreviations

AF: Atrial fibrillation
AFL: Atrial flutter
SGLT2: Sodium-glucose co-transporter 2
HbA1c: Glycated hemoglobin
SBP: Systolic blood pressure
DBP: Diastolic blood pressure
HF: Heart failure
RCT: Randomized controlled trial
RR: Relative risk
CI: Confidence interval
WMD: Weighed mean difference
SD: Standard deviation
PRISRM: Preferred Reporting Items for Systematic Reviews and Meta-analysis
AHRQ: Agency for Healthcare Research and Quality
SOE: Strength of Evidence
RE: Random-effects
FE: Fixed-effects
