# Supplementary material for: SGLT2 inhibitors and atrial fibrillation in type 2 diabetes: a systematic review with meta-analysis of 16 randomized controlled trials
Source: Cardiovasc Diabetol. 2020 Aug 26;19:130. doi: 10.1186/s12933-020-01105-5 (PMC7448518; doi:10.1186/s12933-020-01105-5)

ADDITIONAL FILE

1. Search Algorithm.
2. Sources of data extraction.
3. Additional Figure S1: Forest plot and subgroup meta-analysis of [atrial](javascript:;) [fibrillation](javascript:;)/atrial flutter events.
4. Additional Figure S2: Forest plot and meta-analysis of heart failure.
5. Additional Figure S3: Forest plot and meta-analysis of cerebrovascular events.
6. Additional Figure S4: Forest plot and meta-analysis of m[yocardial](javascript:;) [infarction](javascript:;).
7. Additional Figure S5: Forest plot and meta-analysis of [urinary](javascript:;) [tract](javascript:;) [infection](javascript:;) rate.
8. Additional Figure S6: Forest plot and meta-analysis of adjusted mean HbA1c (%) change from baseline for low dosage.
9. Additional Figure S7: Forest plot and meta-analysis of adjusted mean HbA1c (%) change from baseline for high dosage.
10. Additional Figure S8: Forest plot and meta-analysis of adjusted mean body weight loss change from baseline for low dosage.
11. Additional Figure S9: Forest plot and meta-analysis of adjusted mean body weight loss change from baseline for high dosage.
12. Additional Figure S10: Forest plot and meta-analysis of adjusted SBP change from baseline for low dosage.
13. Additional Figure S11: Forest plot and meta-analysis of adjusted SBP change from baseline for high dosage.
14. Additional Figure S12: Forest plot and meta-analysis of adjusted DBP change from baseline for low dosage.
15. Additional Figure S13: Forest plot and meta-analysis of adjusted DBP change from baseline for high dosage.
16. Additional Figure S14: Random effect meta-regression.
17. Search Algorithm:

Pubmed Search:

(“Type 2 diabetes mellitus”[Mesh] OR “diabetes mellitus type 2”[tiab] OR “diabetes Mellitus, Type 2”[tiab]) AND ( “Dapagliflozin”[tiab] OR “Empagliflozin”[tiab] OR OR “Canagliflozin”[tiab] OR “SGLT2”[tiab] OR “Sodium-glucose co-transporter 2”[tiab]) AND ("Letter"[pt] OR random*[tw] OR “trial”[tiab]) AND (“Atrial fibrillation”[Mesh] OR “Atrial fibrillation” [tiab] OR “all-cause mortality”[Mesh] OR “all-cause mortality”[tiab] OR “cardiovascular death”[Mesh] OR “cardiovascular death”[tiab] OR “MACE”[tiab] OR “major adverse cardiovascular events”[tiab]) NOT (Review[ptyp])

Embase Search:

('diabetes mellitus'/exp OR T2DM:ab,ti) AND (Dapagliflozin:ab,ti OR Empagliflozin:ab,ti OR Ertugliflozin:ab,ti OR Canagliflozin:ab,ti) AND (placebo:ti,ab,de AND random:ab,ti) AND 'controlled study'/de AND ('Atrial fibrillation'/exp OR 'Atrial fibrillation':ab,ti OR 'all-cause mortality'/exp OR 'all-cause mortality':ab,ti OR ' cardiovascular death'/exp OR ' cardiovascular death':ab,ti OR 'major adverse cardiac event'/exp OR 'major adverse cardiovascular event':ab,ti OR 'MACE':ab,ti) NOT ('meta-analysis'/it OR 'animal model'/it OR 'editorial'/it OR 'review'/it OR 'case report':ti)

ClinicalTrials.gov (function ‘Search for Studies’ was used):

(Dapagliflozin OR Empagliflozin OR Canagliflozin OR SGLT2 OR Sodium-glucose co-transporter 2) AND Atrial fibrillation

1. Sources of data extraction.

| Study | Atrial fibrillation/atrial flutter | All-cause mortality | Urinary tract infection | Heart failure | Cerebrovascular events | Myocardial infarction | HbA1c change (%) | Body weight loss change (kg) | Blood presuure change (mm Hg) |  |
| --- | --- | --- | --- | --- | --- | --- | --- | --- | --- | --- |
|  |
| Wilding et al, 2012 | C | M | M | NA | C | NA | C | C | NA |  |
| Bailey et al., 2013 | C | M | M | NA | C | C | M | M | M |  |
| Wilding et al, 2013 | C | NA | M | NA | C | C | C | NA | M |  |
| Kovacs et al., 2014 | C | M | M | NA | NA | NA | M | M | M |  |
| Barnett et al., 2014 | M | M | M | C | C | C | NA | NA | NA |  |
| Yale et al, 2014 | C | M | M | C | C | C | C | NA | NA |  |
| Leiter et al., 2014 | C | M | M | C | C | C | C | C | C |  |
| Bode et al., 2014 | C | M | M | C | C | C | C | NA | M |  |
| Rosenstock et al.,2015 | C | M | M | NA | C | C | M | M | M |  |
| Mathieu et al., 2015 | C | NA | M | M | NA | C | M | M | NA |  |
| NCT00528372(2015) | C | NA | C | C | C | C | NA | NA | NA |  |
| NCT01734785(2016) | C | NA | C | C | NA | NA | C | C | NA |  |
| NCT01989754(2018) CANVAS-R Trial | C | C | C | C | C | C | NA | NA | NA |  |
| NCT01032629(2018) | C | C | C | C | C | C | C | NA | C |  |
| NCT01730534(2019) DARELARE-TIMI58 | C | C | NA | C | C | C | NA | NA | NA |  |
| Perkovic et al, 2019 | C | M | C | M | C | C | NA | NA | NA |  |
| Sources of data extraction from:M--main texts, C--Clinicals.gov, NA-- not available | | | | | | | | | |  |

1. Additional Figure S1: Forest plot and subgroup meta-analysis of atrial fibrillation/atrial flutter events.


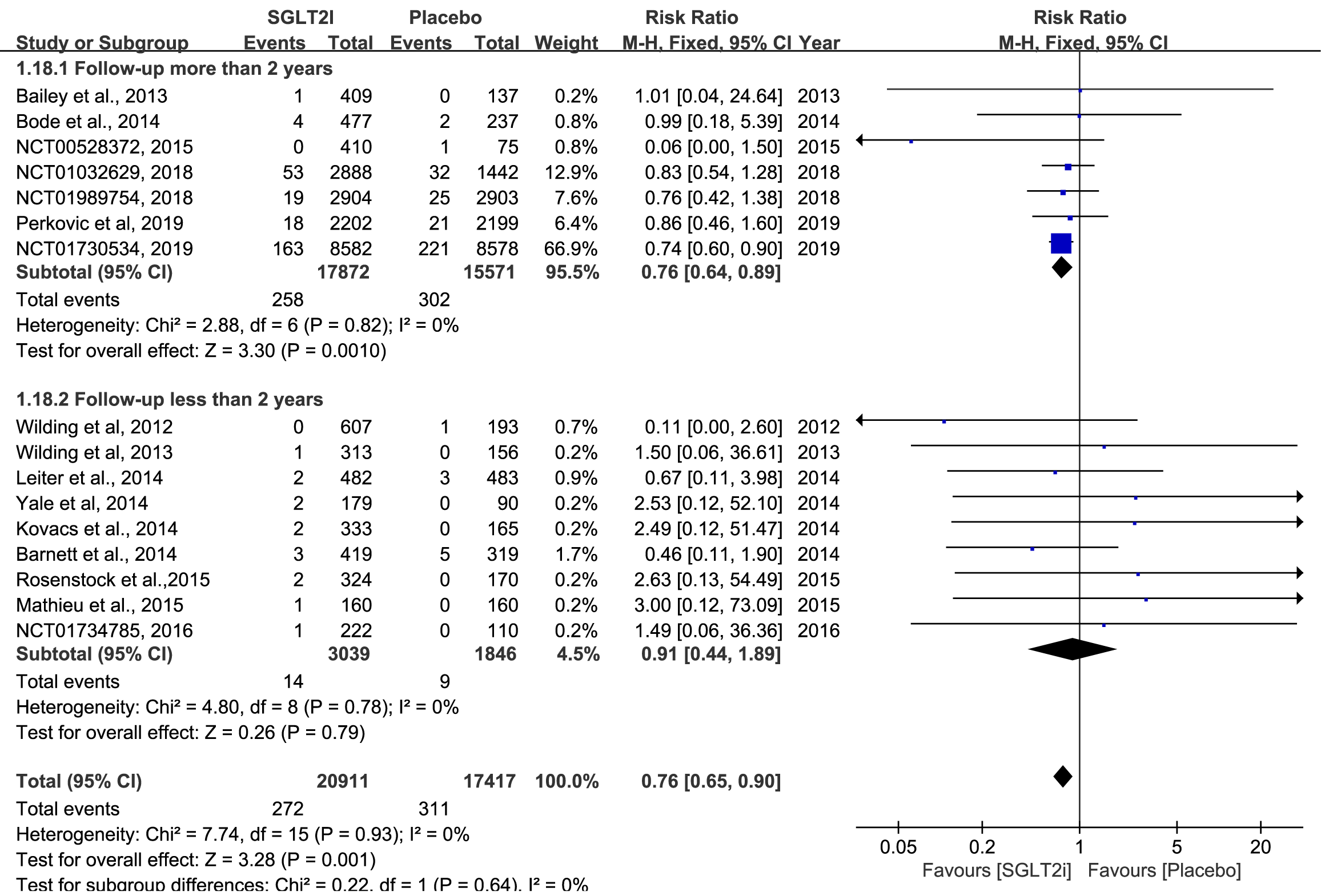


1. Additional Figure S2: Forest plot and meta-analysis of heart failure.


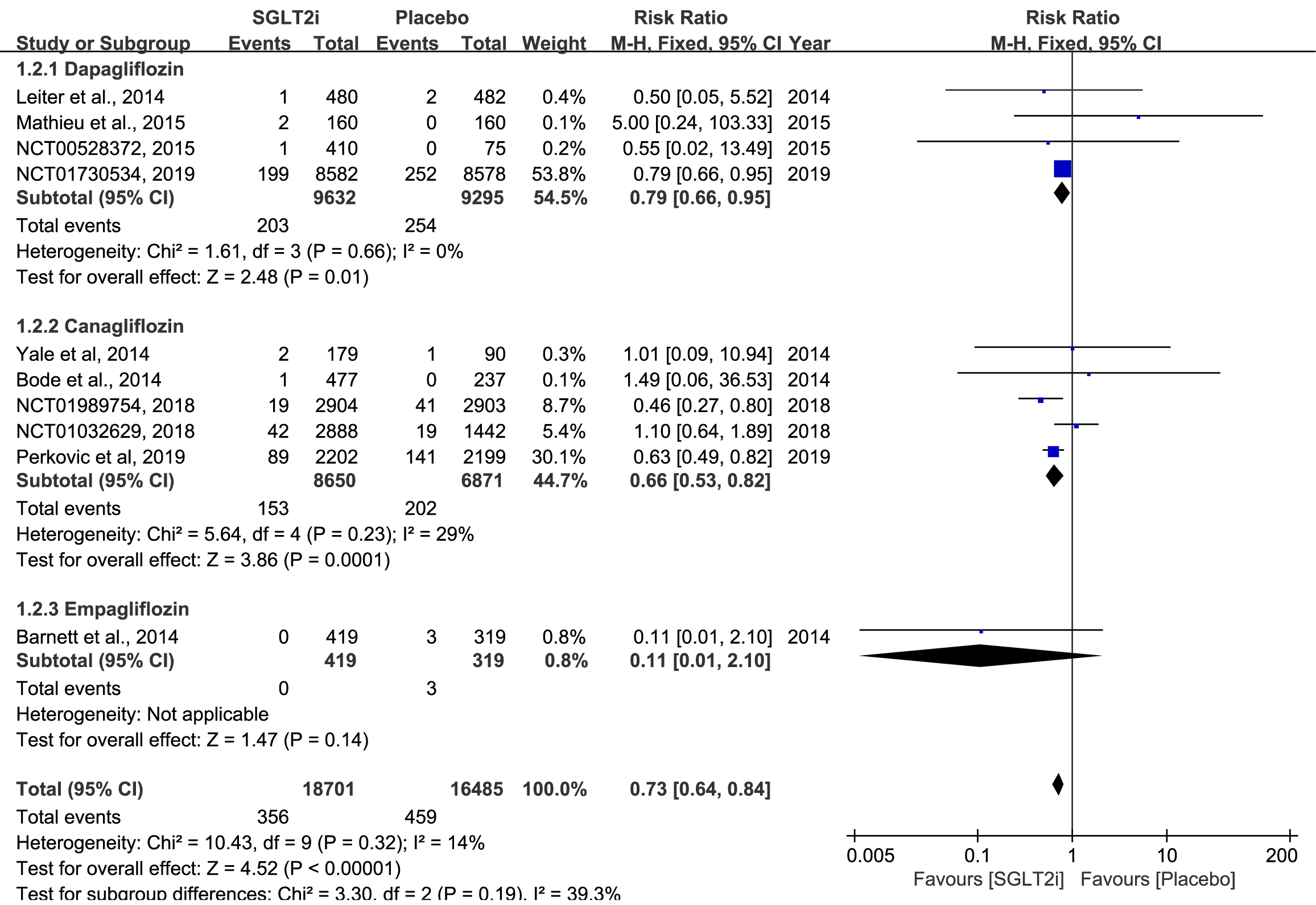


1. Additional Figure S3: Forest plot and meta-analysis of cerebrovascular events.


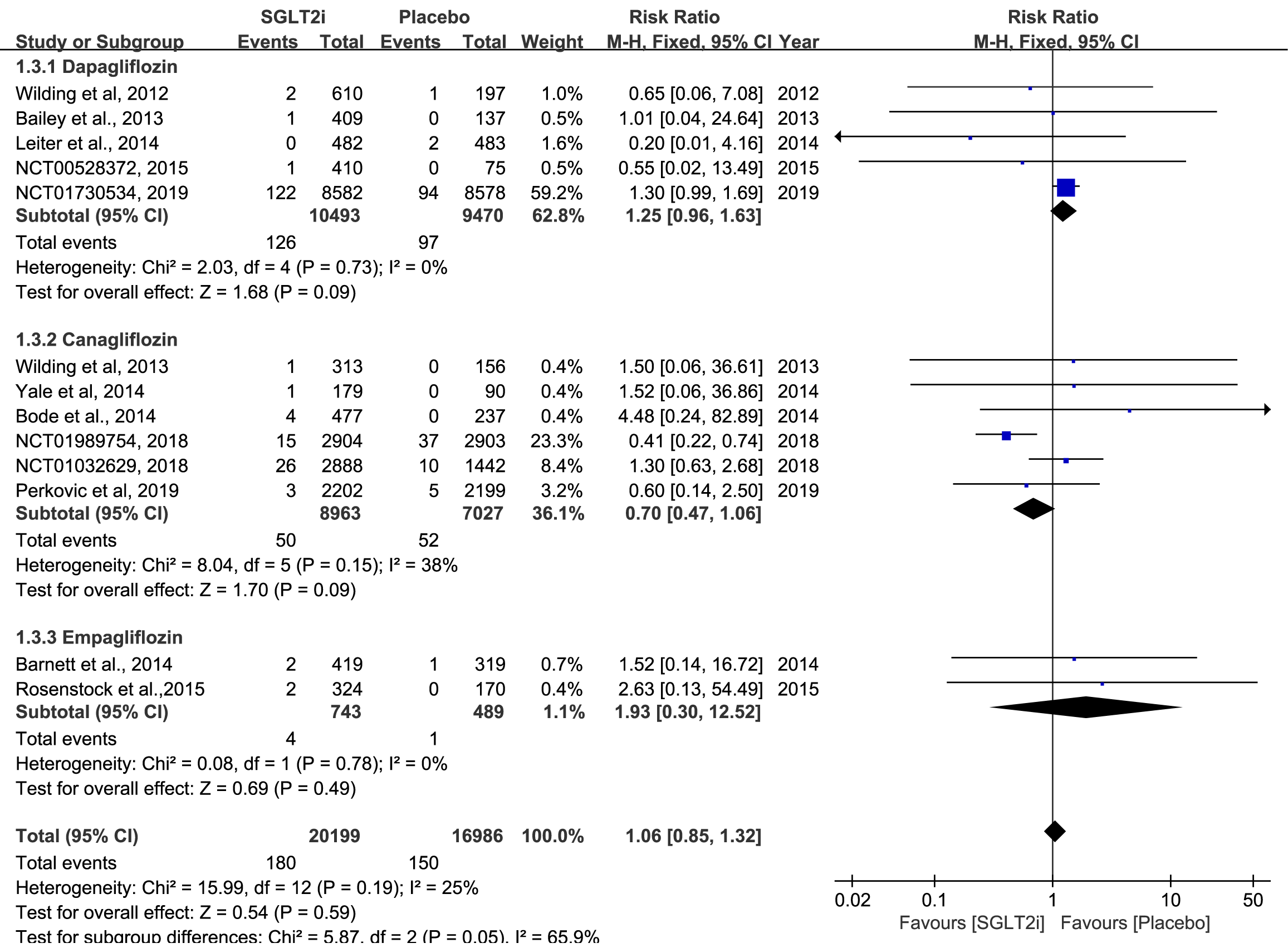


1. Additional Figure S4: Forest plot and meta-analysis of m[yocardial](javascript:;) [infarction](javascript:;).


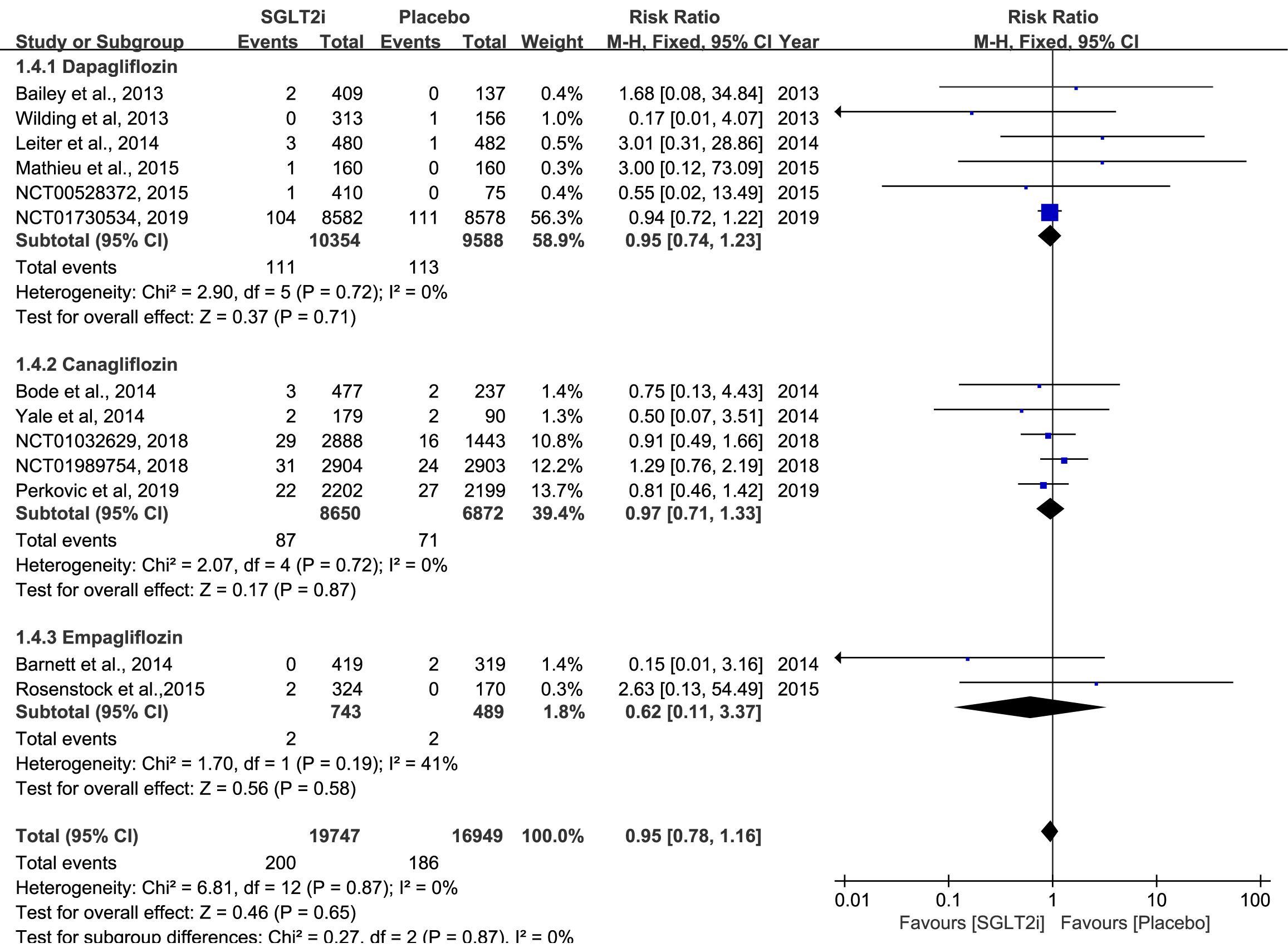


1. Additional Figure S5: Forest plot and meta-analysis of [urinary](javascript:;) [tract](javascript:;) [infection](javascript:;) rate.


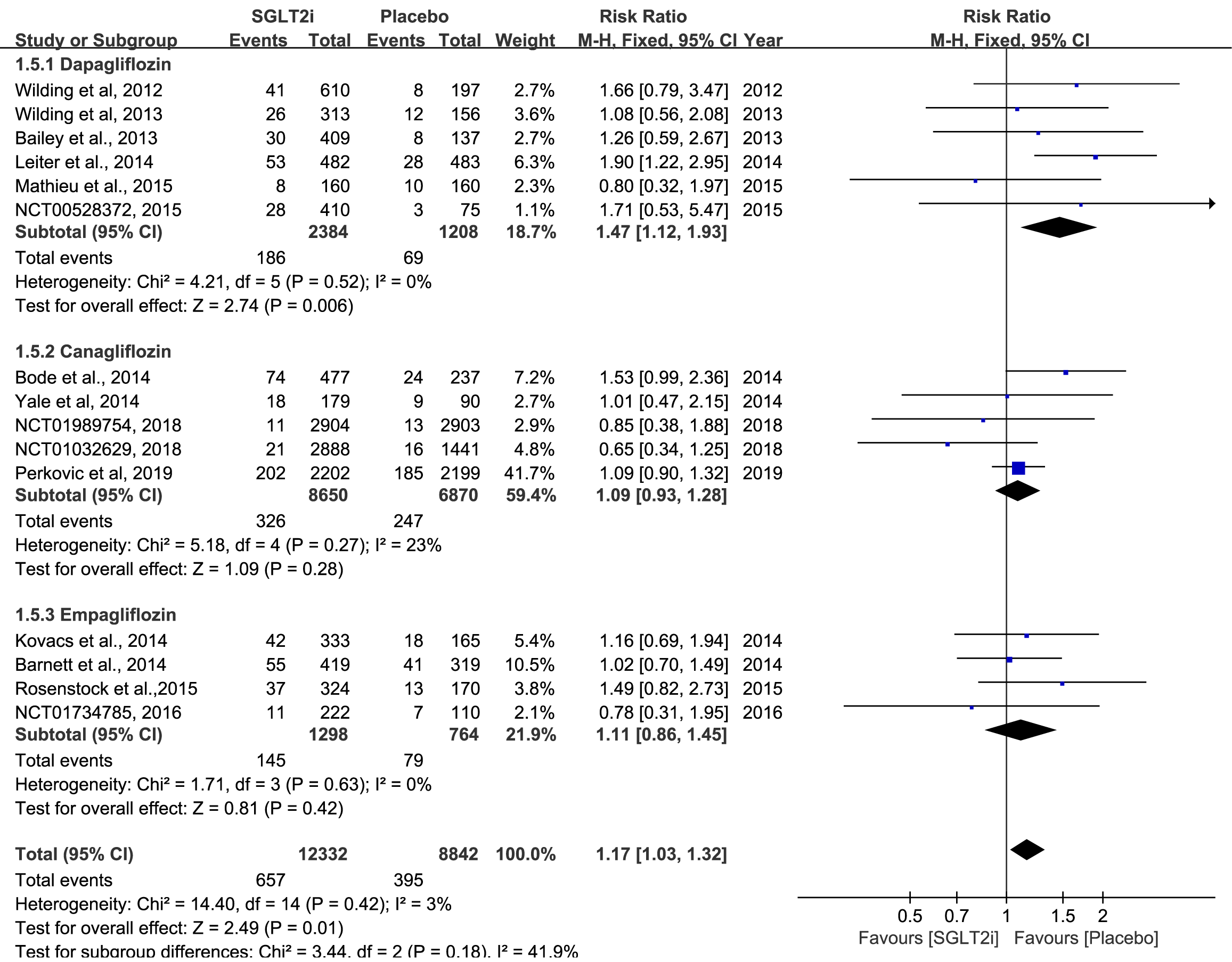


1. Additional Figure S6: Forest plot and meta-analysis of adjusted mean HbA1c (%) change from baseline for low dosage.


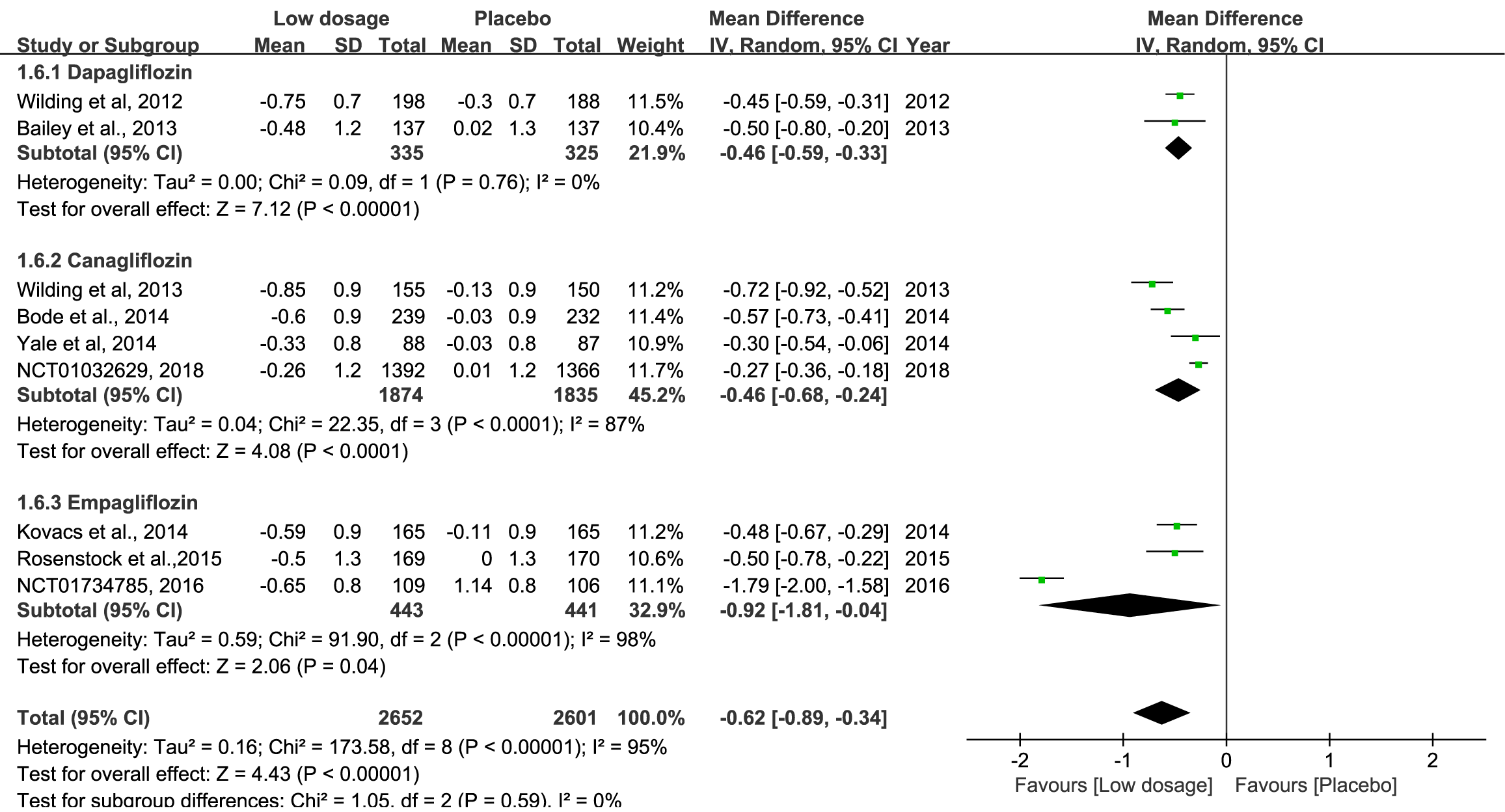


1. Additional Figure S7: Forest plot and meta-analysis of adjusted mean HbA1c (%) change from baseline for high dosage.


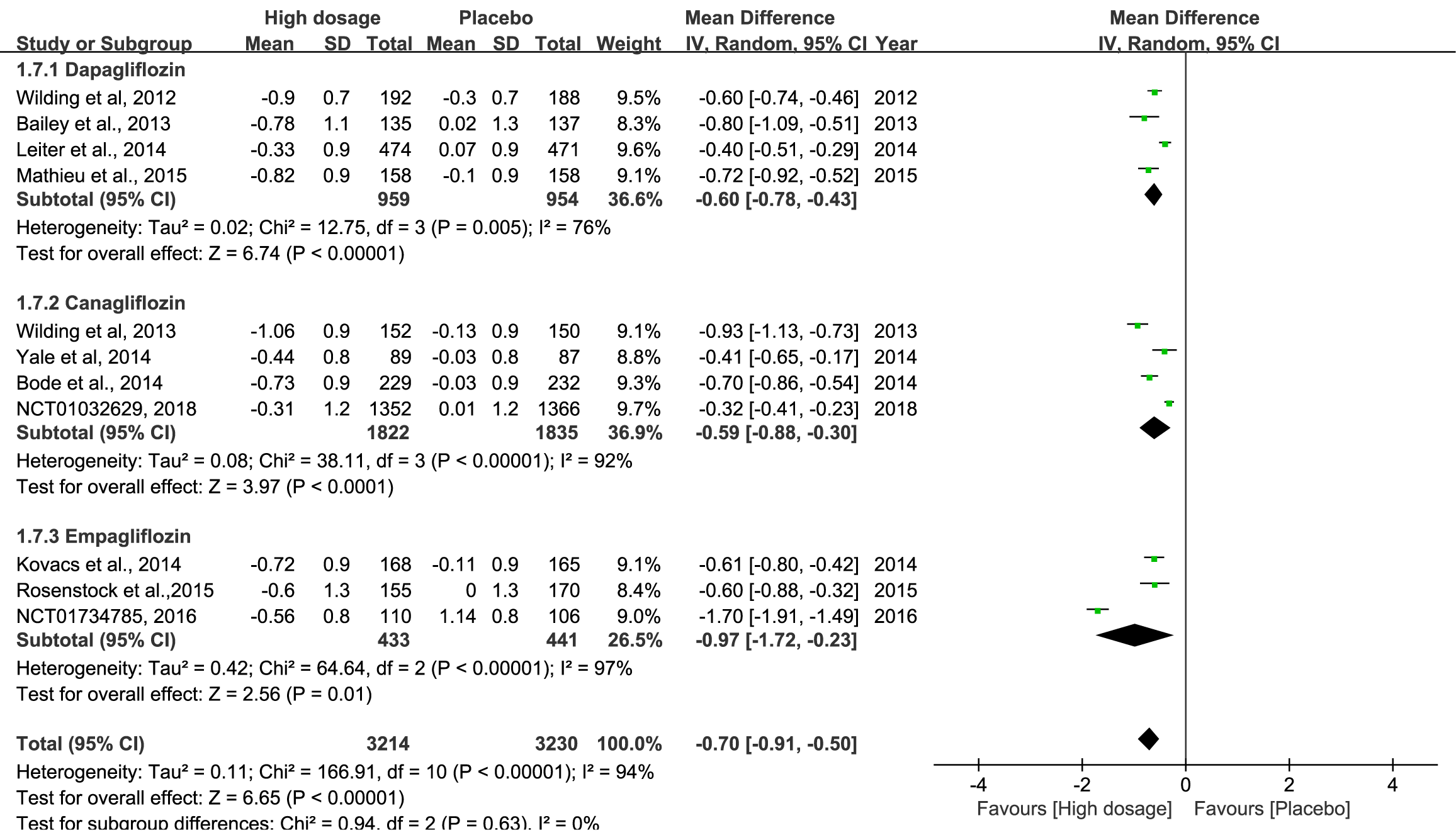


1. Additional Figure S8: Forest plot and meta-analysis of adjusted mean body weight loss change from baseline for low dosage.


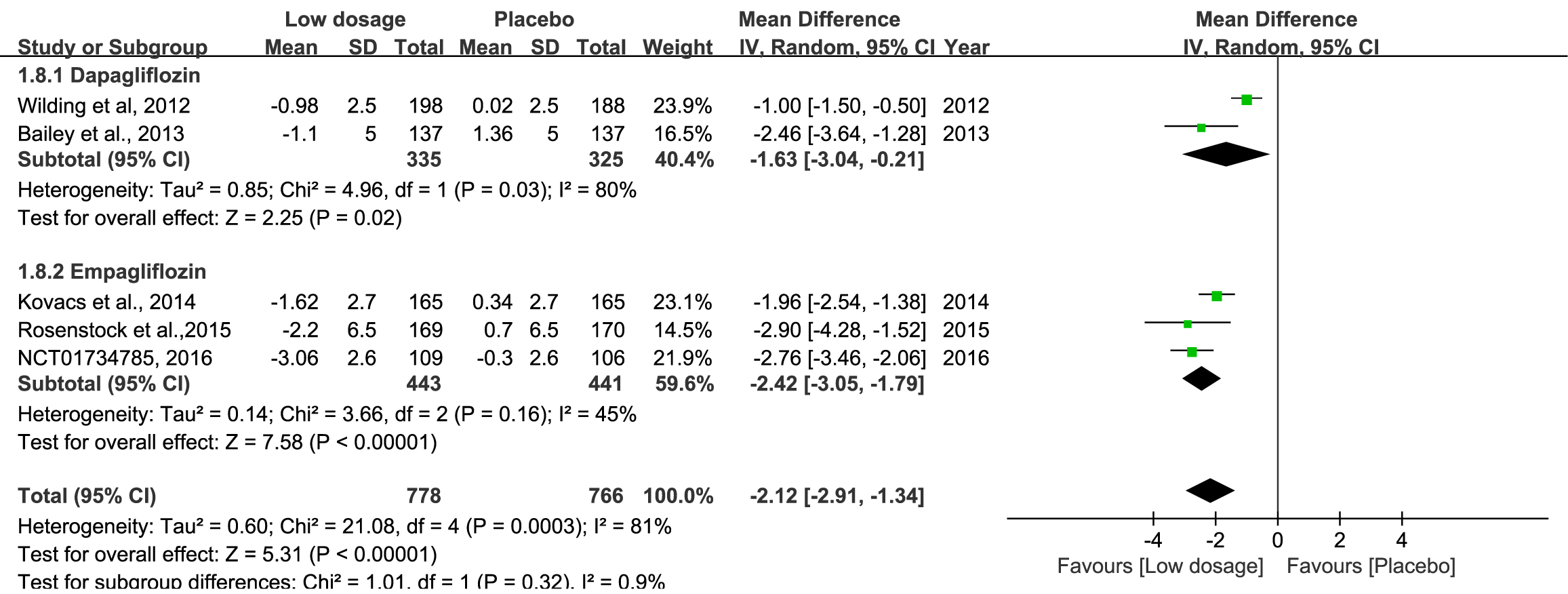


1. Additional Figure S9: Forest plot and meta-analysis of adjusted mean body weight loss change from baseline for high dosage.


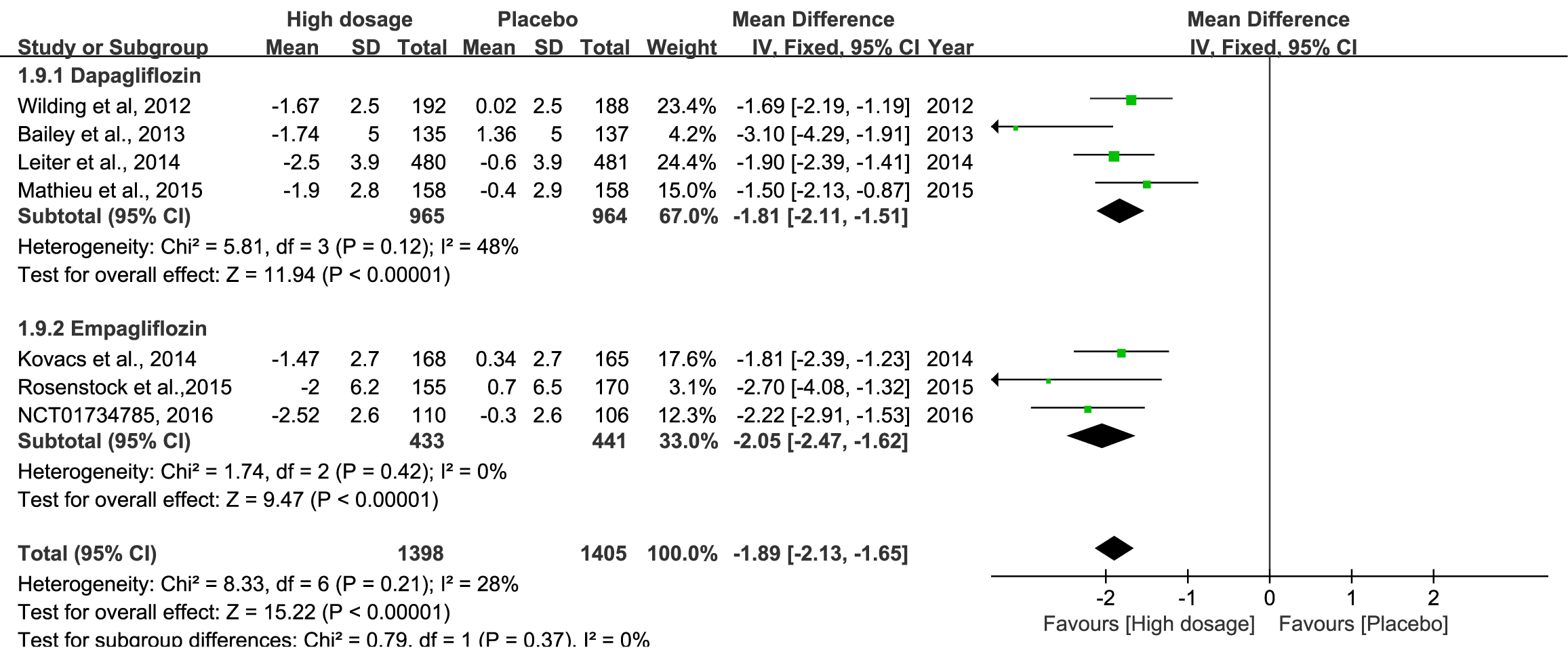


1. Additional Figure S10: Forest plot and meta-analysis of adjusted SBP change from baseline for low dosage.


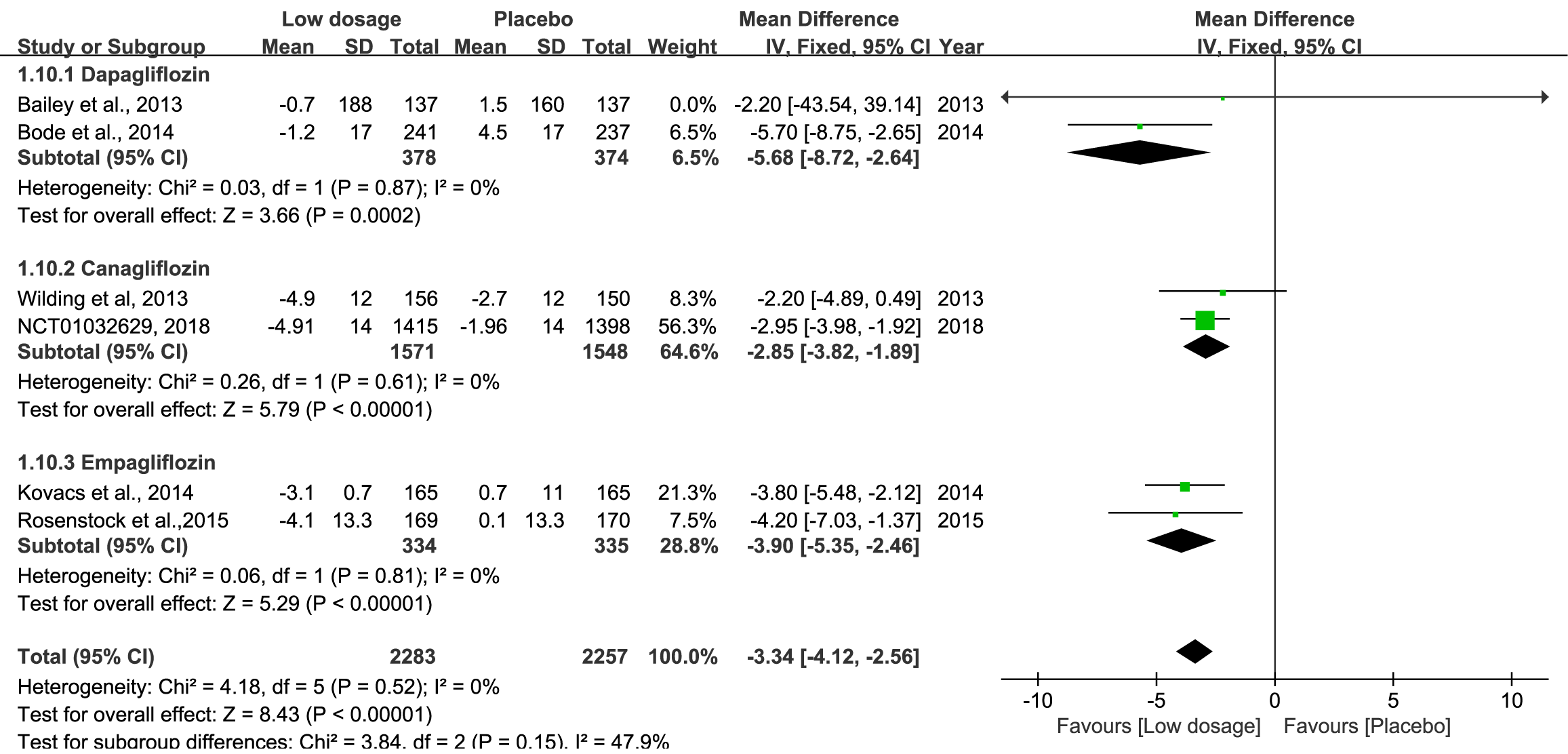


1. Additional Figure S11: Forest plot and meta-analysis of adjusted SBP change from baseline for high dosage.


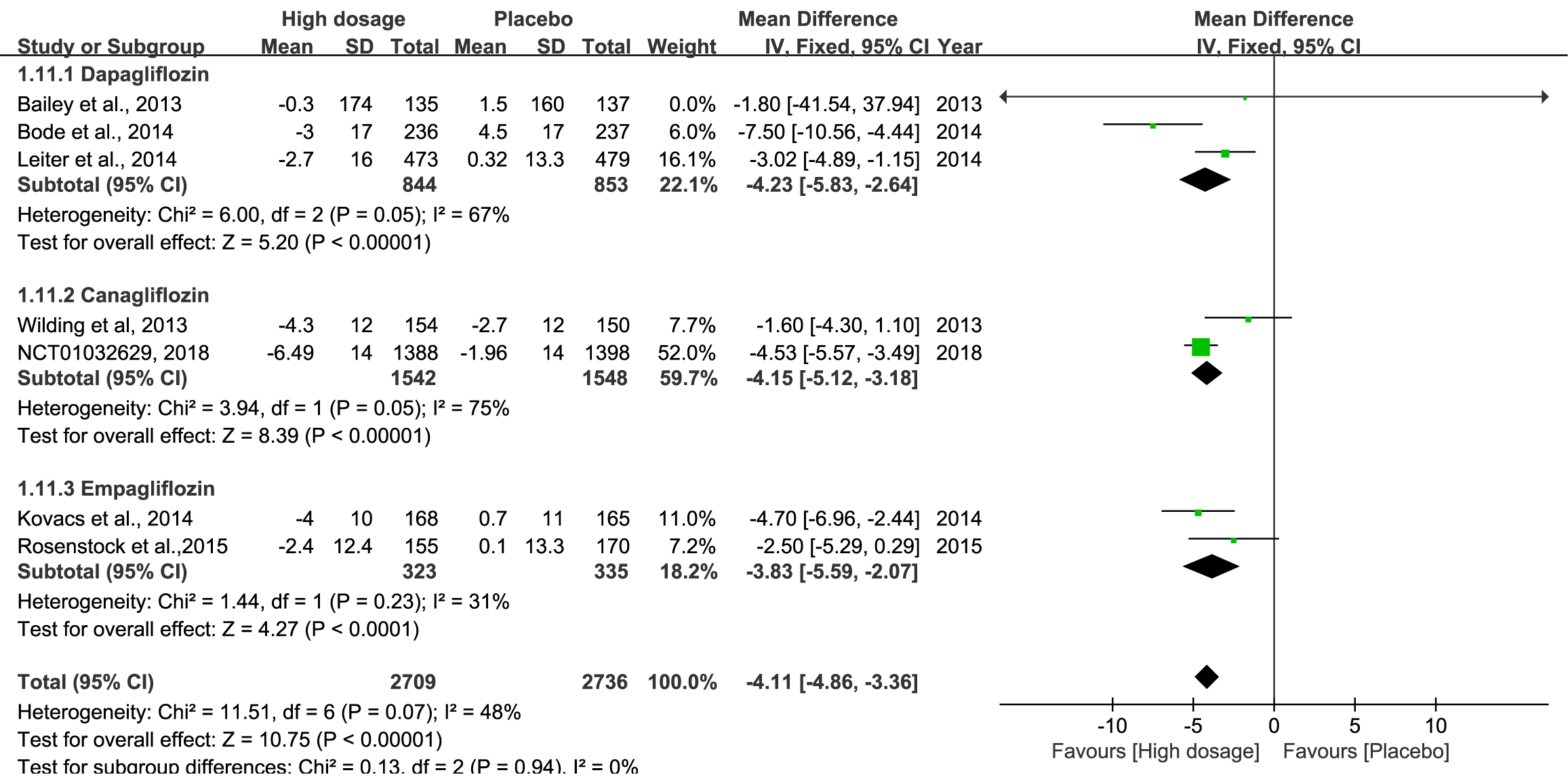


1. Additional Figure S12: Forest plot and meta-analysis of adjusted DBP change from baseline for low dosage.


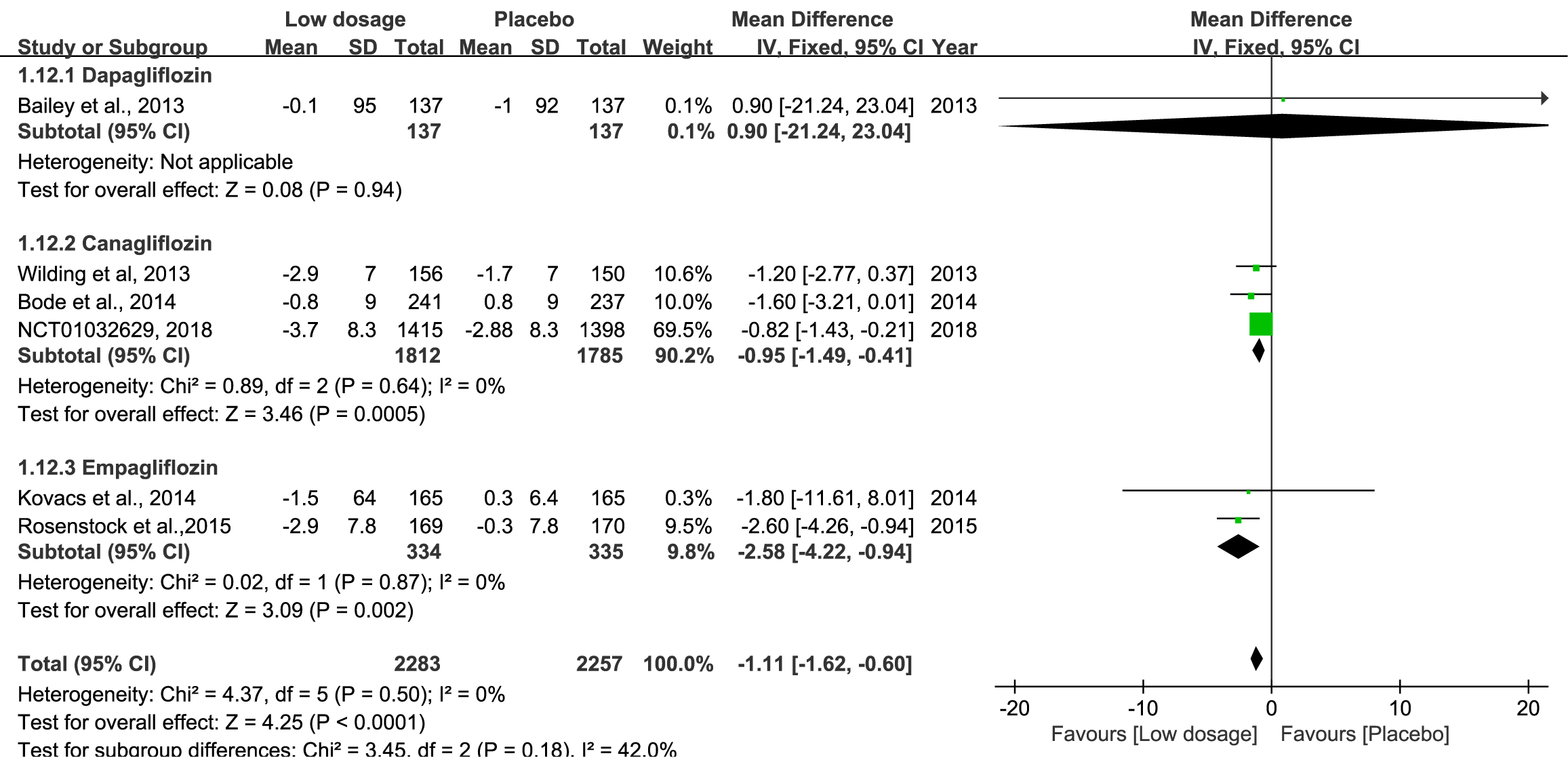


1. Additional Figure S13: Forest plot and meta-analysis of adjusted DBP change from baseline for high dosage.


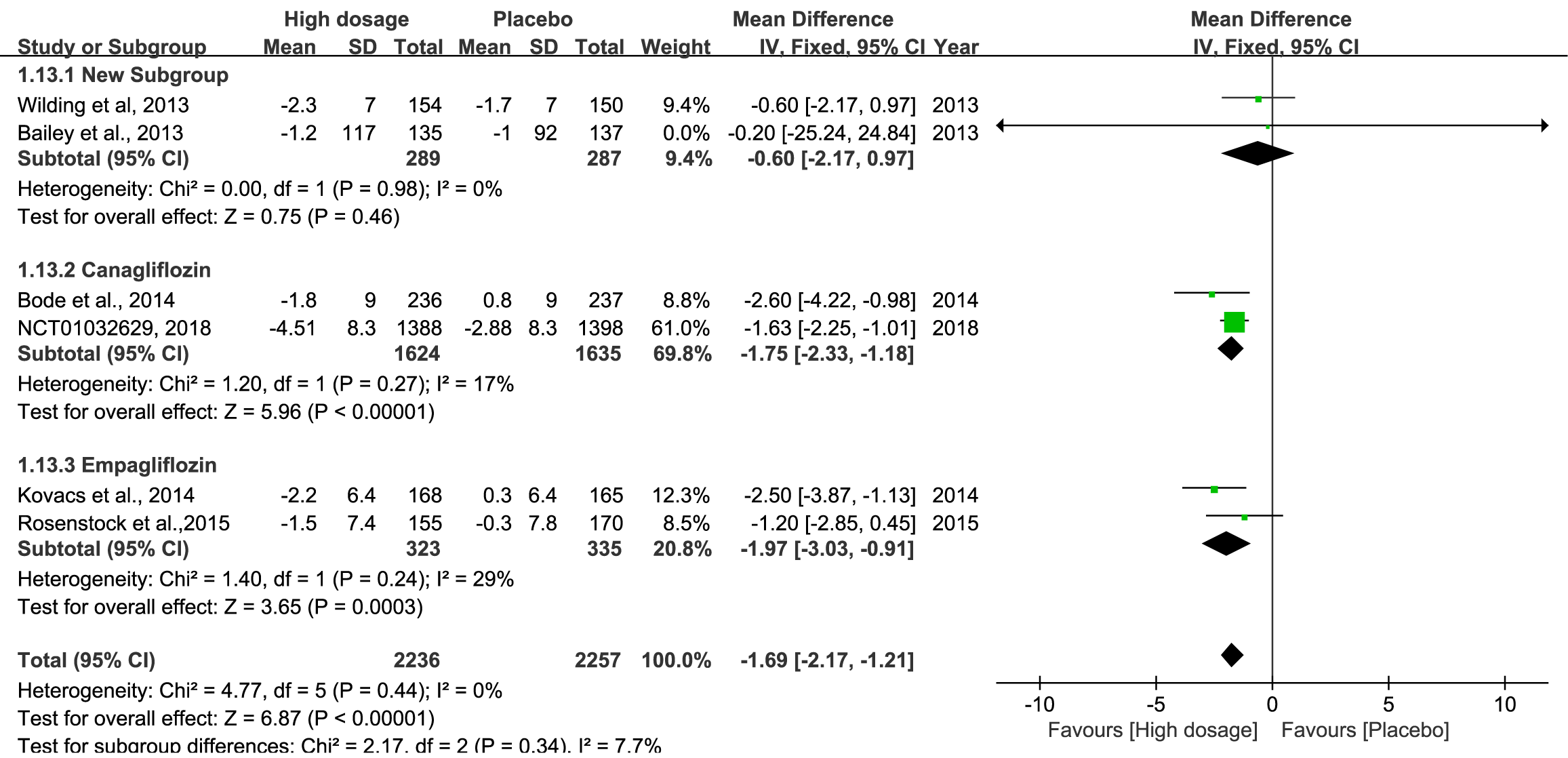


1. Additional Figure S14: Random effect meta-regression.


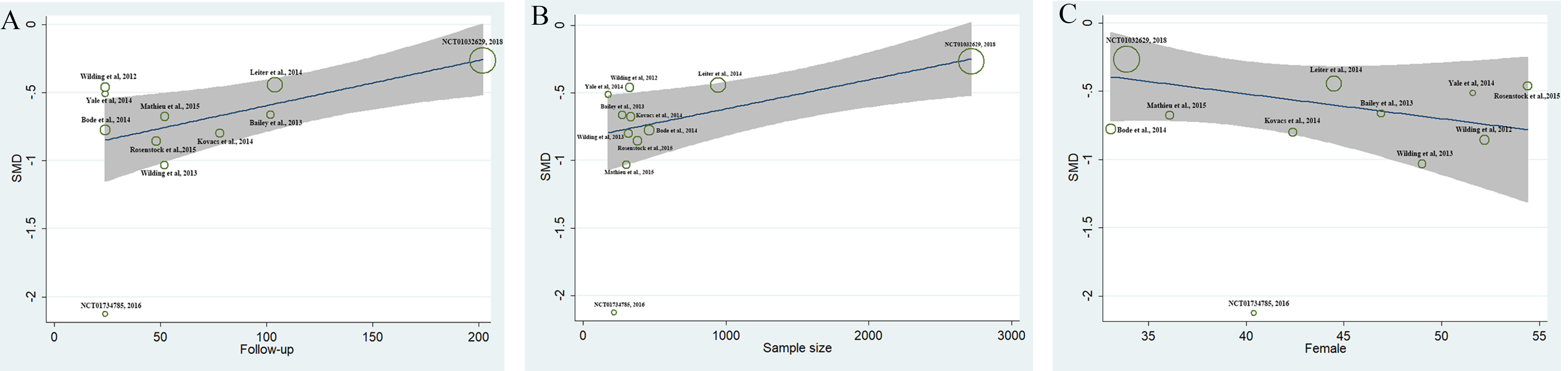

Supplement: Supplementary file 1 — Additional file 1: Figure S1: Forest plot and subgroup meta-analysis of atrial fibrillation/atrial flutter events. Weights are from the fixed-effect analysis. The solid line across the square represents the 95% confidence interval (CI). Figure S2. Forest plot and meta-analysis of heart failure. Weights are from the fixed-effect analysis. The solid line across the square represents the 95% confidence interval (CI). Figure S3. Forest plot and meta-analysis of cerebrovascular events. Weights are from the fixed-effect analysis. The solid line across the square represents the 95% confidence interval (CI). Figure S4. Forest plot and meta-analysis of myocardial infarction. Weights are from the fixed-effect analysis. The solid line across the square represents the 95% confidence interval (CI). Figure S5. Forest plot and meta-analysis of urinary tract infection rate. Weights are from the fixed-effect analysis. The solid line across the square represents the 95% confidence interval (CI). Figure S6: Forest plot and meta-analysis of adjusted mean HbA1c change from baseline for low dosage. Weights are from the random-effect analysis. The solid line across the square represents the 95% confidence interval (CI). Figure S7. Forest plot and meta-analysis of adjusted mean HbA1c change from baseline for high dosage. Weights are from the random-effect analysis. The solid line across the square represents the 95% confidence interval (CI). Figure S8. Forest plot and meta-analysis of adjusted mean body weight loss change from baseline for low dosage. Weights are from the random-effect analysis. The solid line across the square represents the 95% confidence interval (CI). Figure S9. Forest plot and meta-analysis of adjusted mean body weight loss change from baseline for low dosage. Weights are from the random-effect analysis. The solid line across the square represents the 95% confidence interval (CI). Figure S10. Forest plot and meta-analysis of adjusted mean SBP change from baseline for [file 12933_2020_1105_MOESM1_ESM.doc]
